# Pharmacokinetic Relevance of In Vitro Skin Models: Absorption, Cutaneous Distribution, Metabolism, and Clearance-like Removal in Dermal and Transdermal Drug Testing

**DOI:** 10.3390/pharmaceutics18091152

**Published:** 2026-09-14

**Authors:** Filip Dugonik, Urška Jeršič, Lejla Kač, Uroš Maver, Tina Maver

**Affiliations:** 1 Institute of Biomedical Sciences, Faculty of Medicine, University of Maribor, Taborska ulica 8, 2000 Maribor, Slovenia; filip.dugonik@student.um.si (F.D.); urska.jersic@student.um.si (U.J.); lejla.kac@student.um.si (L.K.); 2 Department of Pharmacology, Faculty of Medicine, University of Maribor, Taborska ulica 8, 2000 Maribor, Slovenia

**Keywords:** in vitro skin models, dermatopharmacokinetics, dermal drug delivery, transdermal drug delivery, in vitro permeation testing, fit-for-purpose model selection

## Abstract

**Background**: In vitro skin models are widely used in dermal and transdermal drug development, yet their pharmacokinetic interpretation often remains endpoint-dependent and does not fully capture cutaneous disposition, including partitioning, local metabolism, or clearance-like removal. This review evaluates the utility of these models through a comprehensive pharmacokinetic process framework. **Methods**: Using a structured literature search, we critically compared data from studies utilizing a range of in vitro skin models, including synthetic membranes, ex vivo human skin, reconstructed human epidermis, full-thickness equivalents, and advanced bioprinted or microfluidic platforms. The assessment focused on model performance across the key processes of absorption, distribution, metabolism, and elimination. **Results**: Our analysis reveals that alternative barriers exhibit no uniform direction of bias relative to human skin; model rank order varies with compound, formulation, and dose, so that the available evidence does not currently support a single universal scaling relationship, although calibration within a defined applicability domain may remain possible. Synthetic membranes provide reproducible platforms for formulation ranking but lack biological retention and metabolism. Ex vivo human skin remains the closest reference for permeation and mass balance, though donor variability and storage sensitivity limit standardization. Reconstructed epidermis supports standardized permeation and epidermal targeting, while full-thickness equivalents permit dermal retention analysis. Advanced perfused constructs extend experimental capabilities, but their predictive performance remains largely uncharacterized. **Conclusions**: No single model universally replicates human pharmacokinetics. Model selection must therefore be carefully aligned with the specific pharmacokinetic process of interest, the model’s biological capacity, and the intended context of use to ensure clinically relevant data interpretation.

## 1. Introduction

Dermal and transdermal drug development requires experimental models that can distinguish between drug permeation across the skin, local tissue retention, systemic absorption, and, where relevant, cutaneous metabolism. This distinction is essential because topical and transdermal products are not always designed to achieve the same pharmacokinetic outcome: some aim to maximize local exposure within the epidermis or dermis, whereas others are intended to deliver the drug into the systemic circulation. Therefore, interpretation of in vitro skin data requires more than measurement of cumulative permeation into a receptor phase.

Skin pharmacokinetics is governed by multiple sequential and interacting processes. After topical application, a compound may partition into the stratum corneum, diffuse through epidermal and dermal compartments, undergo local biotransformation, accumulate within tissue reservoirs, or reach a receptor-side or vascular compartment. The pharmacokinetic relevance of an in vitro skin model, therefore, depends not simply on whether it permits permeation, but on which components of absorption, distribution, metabolism, and clearance it can reproduce [1,2].

A broad spectrum of experimental systems is currently used for dermal and transdermal testing, including synthetic membranes, ex vivo human and animal skin, reconstructed human epidermis, full-thickness skin equivalents, 3D-bioprinted skin, and perfused skin-on-chip platforms. Such systems are increasingly framed as non-animal methods for dermal drug development and safety assessment [3]. These models differ substantially in biological complexity, barrier maturity, cellular viability, tissue compartmentalization, metabolic competence, and ability to reproduce clearance-like removal. Consequently, no single model can capture the full pharmacokinetic behavior of human skin. Synthetic membranes provide reproducible physicochemical barriers for early absorption screening, ex vivo human skin remains the closest conventional reference for permeation and tissue retention, reconstructed human skin models offer standardized human-cell-based platforms, and perfused or bioprinted systems increasingly enable investigation of dynamic transport and spatially defined tissue architecture [4,5,6,7,8].

**Figure 1:**
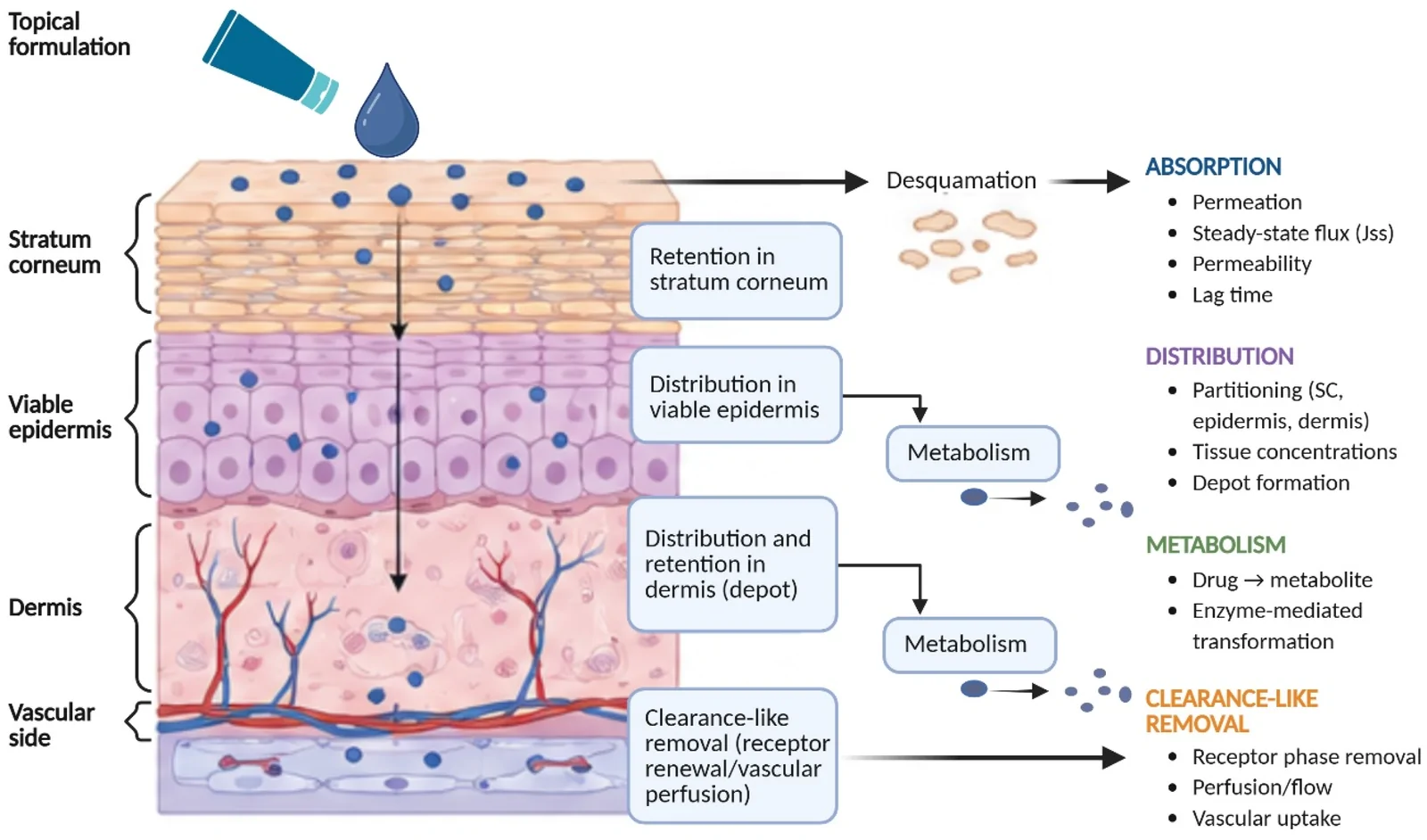
Pharmacokinetic disposition of a topically applied compound and the corresponding process-specific endpoints accessible to in vitro skin models. “Pharmacokinetic disposition of a topically applied compound and the corresponding process-specific endpoints accessible to in vitro skin models”created in Biorender. Maver, U. (https://BioRender.com/6jzq8zp) is licensed under CC BY 4.0.

This review evaluates the pharmacokinetic relevance of in vitro skin models through an absorption, distribution, metabolism, and clearance framework (Figure 1). Rather than ranking models by structural complexity alone, we compare what each platform can and cannot reveal about permeability, flux, lag time (tlag), tissue retention, local metabolism, receptor-side transport, and clearance-like removal.

The aim is to support the selection of fit-for-purpose models in dermal and transdermal drug development, formulation comparison, toxicological assessment, and regulatory translation. We therefore distinguish four dimensions of evidence relevant to model use that should not be conflated. Biological capability refers to the structural and functional processes represented by the model, including barrier architecture, viable tissue compartments, appendages, metabolic competence, and, where present, a perfused vascular-like compartment. Analytical performance refers to the reliability of the measurement chain used to generate the endpoint, rather than to the biological complexity of the model or to quantitative synthesis of the literature. It includes adequate analytical sensitivity relative to the expected concentrations, validated recoveries from the analyzed matrices, compound stability, matrix effects, and the contribution of analytical recovery to the overall mass balance assessment. Predictive agreement refers to a quantitative comparison with a predefined reference preparation under matched experimental conditions. For claims of human-relevant skin permeation and retention, ex vivo human skin is treated as the principal conventional reference comparator in this review. Organization for Economic Co-operation and Development (OECD) Test Guideline (TG) 428 and OECD Guidance Document 156 permit excised human or animal skin, whereas current U.S. Food and Drug Administration (FDA) and European Medicines Agency (EMA) in vitro permeation testing (IVPT) guidance for topical equivalence centers on ex vivo human skin [9,10,11,12]. In vivo human data remain important for in vitro–in vivo extrapolation (IVIVE) and translational assessment, but are not treated here as a direct tissue reference because they also reflect systemic circulation, extracutaneous distribution, metabolism, and clearance. Regulatory qualification refers to documented validation or regulatory acceptance of a model or method for a specified endpoint and context of use.

A model may perform strongly in one dimension and weakly in another, and the relative importance of these dimensions depends on the intended application. For early formulation ranking, robust analytical performance and repeatability may be sufficient without requiring absolute quantitative agreement with human skin or formal regulatory qualification. When an alternative model is intended to support quantitative human-relevant permeation or retention claims, matched benchmarking against ex vivo human skin becomes important. For regulatory use, qualification must additionally be demonstrated for the specific endpoint and context of use and should not be assumed to transfer between applications. Accordingly, the supporting evidence is dimension-specific: structural and functional characterization for biological capability; validated analytical sensitivity and recovery for analytical performance; matched-condition comparison with predefined agreement criteria for predictive agreement; and appropriate validation studies, test guidelines, or regulatory guidance for regulatory qualification.

**Figure 2:**
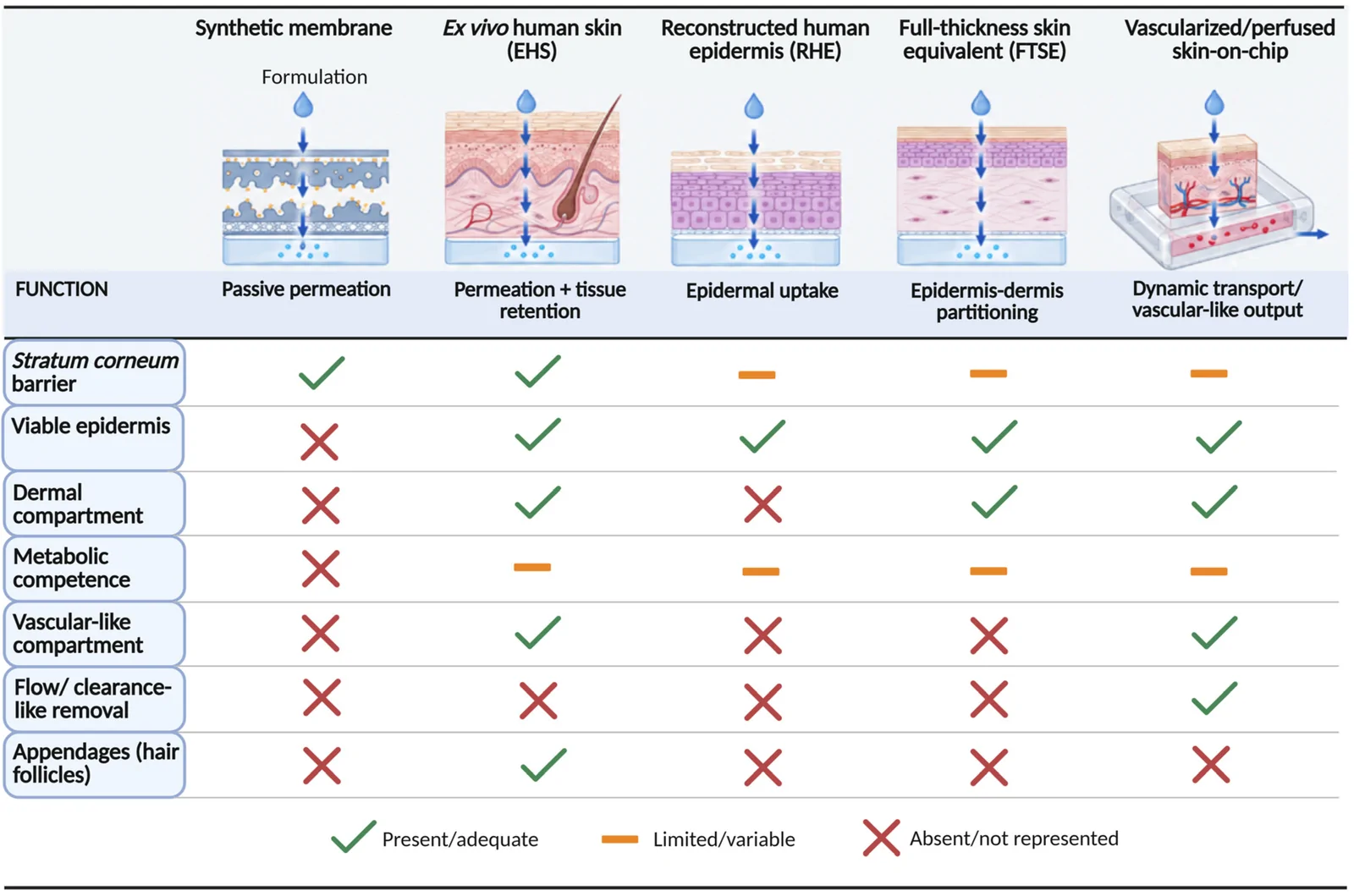
Principal classes of in vitro skin models and the structural and functional features they represent: a synthetic membrane (Strat-M^®^), excised ex vivo human skin (EHS), reconstructed human epidermis (RHE), a full-thickness skin equivalent (FTSE), and a vascularized or perfused skin-on-chip system. The upper schematics illustrate the principal experimental transport pathway and dominant functional use of each model class, including topical formulation application, compound transport across the barrier, and the main downstream disposition context. The functional labels in Figure 2 indicate the principal use highlighted for each model class and are not intended to represent exclusive capabilities. The lower capability matrix indicates whether a component or function is present and adequately represented (green check), present but limited or variable between models, batches, or protocols (orange minus), or absent/not represented (red cross). Symbols denote representation of model features rather than accuracy of quantitative prediction: distinct architectures confer distinct capabilities, and increasing structural complexity does not imply a hierarchy of predictive validity. For EHS, metabolic competence is storage-dependent: fresh viable tissue retains broader metabolic activity, whereas frozen/thawed skin may retain residual hydrolytic activity, particularly esterase-mediated hydrolysis, despite reduced viability-dependent oxidative and conjugative metabolism. Appendageal structures include hair follicles and sweat ducts; in advanced engineered models, appendage-like structures remain limited or experimental and have not yet been established as functional penetration pathways. “Principal classes of in vitro skin models and the structural and functional features they represent: a synthetic membrane (Strat-M^®^), excised ex vivo human skin (EHS), reconstructed human epidermis (RHE), a full-thickness skin equivalent (FTSE), and a vascularized or perfused skin-on-chip system”created in BioRender. Maver, U. (https://BioRender.com/b7l6tn1) is licensed under CC BY 4.0.

Figure 2 summarizes both the principal transport pathway and the structural and functional features of each model class, while emphasizing that the presence of a compartment or process does not, by itself, establish quantitative predictive performance for the corresponding endpoint.

### Scope and Literature Search Strategy

This review was designed as a critical narrative review with a structured literature search. Several recent reviews have described the technological evolution of in vitro skin models from static membranes through reconstructed tissues to microphysiological systems and have characterized the barrier limitations of human skin equivalents relative to native tissue [1,5]. These reviews have largely organized the field according to model architecture and technological complexity. Here, we adopt a complementary, process-centered pharmacokinetic framework: rather than asking how complex a model is, we evaluate which aspects of cutaneous drug disposition can meaningfully be reproduced or resolved. This process-first perspective allows a structurally simple model to be considered appropriate for one pharmacokinetic endpoint while remaining inadequate for another.

To identify relevant evidence, PubMed/MEDLINE, Scopus and Web of Science were searched from database inception to August 2026 using combinations of the terms “in vitro permeation”, “IVPT”, “Franz diffusion cell”, “skin absorption”, “Strat-M”, “reconstructed human epidermis”, “full-thickness skin equivalent”, “bioprinted skin”, “skin-on-chip”, “dermatopharmacokinetics”, “cutaneous metabolism” and “dermal clearance”, combined with “permeability coefficient”, “flux”, “lag time”, “tissue retention” and “mass balance”. Priority was given to primary studies directly comparing two or more barrier types under matched experimental conditions, formal prevalidation and validation studies, and relevant regulatory guidance issued by the OECD, EMA, and FDA. Studies were considered when they reported experimental endpoints relevant to absorption, cutaneous distribution or retention, metabolism, transporter activity, or clearance-like transport, or when they contributed evidence relevant to model validation, IVIVE, physiologically based pharmacokinetic (PBPK) modeling, or regulatory application. Studies that address skin models exclusively for regenerative medicine, wound healing, or disease modeling, without a pharmacokinetic or barrier-transport endpoint, were outside the scope of this review.

Given the substantial heterogeneity in model architecture, permeants, formulations, dose regimens, tissue preparation, receptor conditions, sampling schedules, and analytical recovery, the literature was synthesized qualitatively and mechanistically rather than quantitatively. This review was not conducted as a systematic review: no formal screening flow was applied, no formal risk-of-bias assessment or meta-analysis was performed, and the review protocol was not registered. The literature search was structured around predefined databases and search concepts, but study selection and synthesis were conducted as a critical narrative review rather than an exhaustive systematic assessment. Reported permeability coefficients (Kp), fluxes, tlag, and tissue-retention values were retained as presented in the original publications rather than recalculated or normalized across experimental conditions. Quantitative values should therefore be interpreted together with the dose regimen, receptor conditions, and tissue preparation under which they were generated and are used here primarily to illustrate model-dependent behavior rather than to establish universal quantitative relationships between model systems.

## 2. Pharmacokinetic Endpoints and Methodological Determinants of Dermal and Transdermal Testing

### 2.1. Absorption, Distribution, Metabolism, and Clearance-like Removal

The pharmacokinetic interpretation of in vitro skin models depends on the endpoint being measured. In dermal and transdermal testing, receptor-phase permeation is only one part of drug disposition. A topically applied compound may remain on the surface, partition into the stratum corneum, accumulate in viable epidermis or dermis, undergo local metabolism, or, in perfused systems, reach a receptor-side or vascular-like compartment. Model performance should therefore be evaluated according to the pharmacokinetic process of interest rather than by structural complexity alone [1,2].

The most reported absorption endpoints are cumulative amount permeated, steady-state flux (Jss), maximum flux, apparent Kp, and tlag. Cumulative permeation refers to the amount of drug recovered in the receptor phase over time, typically normalized to the exposed area and corrected for drug removed with preceding samples (Box 1, Equation (1)). Jss reflects the rate of transport during the linear permeation phase (Box 1, Equation (2)), whereas the apparent Kp relates steady-state flux to the concentration of compound dissolved in the donor phase (Box 1, Equation (3)). Where the donor is applied neat or at saturation, that is, at maximum thermodynamic activity, the corresponding upper bound of flux attainable from that vehicle is Jmax (Box 1, Equation (4)). Tlag indicates the delay before receptor-phase appearance and is influenced by barrier thickness, diffusion path length, tissue partitioning, and formulation behavior. These endpoints are strongly affected by donor dose, vehicle composition, receptor medium composition, sink conditions, tissue thickness, temperature, stirring, and sampling frequency [2,13]. Jss and the apparent Kp are therefore conditional endpoints and should only be reported when donor activity remains approximately constant, receptor-side sink conditions are maintained, and a genuine linear permeation interval is demonstrated; apparent tlag likewise requires a clearly defined linear post-lag phase (Section 2.2). Under finite-dose conditions, cumulative amount permeated, peak flux (Jpeak, together with tpeak; Box 1, Equation (5)), and mass balance are generally more appropriate descriptors.

Box 1 **Definitions, equations, and units of the principal permeation endpoints.**

The absorption endpoints introduced above are defined below with their equations and units. Two of them are conditional rather than universal: Jss and the apparent Kp are defined only where donor activity is approximately constant and receptor-side sink conditions are maintained, so that a genuine linear permeation interval exists (Section 2.2) [14,15].

**Cumulative amount permeated per unit area, Q(t)—µg cm^−2^**

       The total amount of compound recovered in the receptor phase up to time t, divided by the exposed diffusion area. Where the receptor phase is sampled and replaced, the cumulative amount must be corrected for the drug removed with all preceding samples [16].

(1) $${Q\left(t\right)}_{n}=\frac{\left[C_{n}V_{r}+\sum_{i=1}^{n-1}C_{i}V_{s}\right]}{A}$$

where C*n* is the measured receptor concentration at sampling time n, V*r* the receptor volume, C*i* the concentration at each preceding sampling time i, V*s* the sample volume withdrawn and replaced, and A the exposed diffusion area (cm^2^). Reporting uncorrected concentrations progressively understates cumulative permeation at each sampling point.

**Steady-state flux, Jss—µg cm^−2^ h^−1^ (conditional)**

The slope of the cumulative amount permeated against time over the linear portion of the profile [15].

(2) $$J_{ss}=\frac{dQ}{dt}$$

evaluated over the interval in which dQ/dt is constant. The time window used and the goodness of fit should be reported, since the demonstrated linearity of that interval is what justifies the term steady state. Including data collected before steady state underestimates both Kp and tlag, and the experiment should therefore run for at least approximately three lag times; objective, algorithmic determination of the steady-state boundaries has been proposed as an alternative to visual selection [14,17,18].

**Apparent permeability coefficient, Kp—cm h^−1^ (conditional)**

The Jss normalized to the donor concentration [15,16].

(3) $$K_{p}=\frac{J_{ss}}{C_{d}}$$

where Cd is the concentration of compound dissolved in the donor phase and in contact with the barrier over the interval used to determine Jss. Under infinite-dose conditions, Cd is commonly approximated by the initial donor concentration C0, but this approximation holds only where the donor phase is demonstrably not depleted, and its composition does not change [15]. Cd should therefore be the measured, dissolved concentration rather than the nominal concentration; for saturated suspensions, the solubility of the compound in the vehicle should be used and the degree of saturation reported; a nominal concentration is acceptable only where it has been verified analytically. Because Kp is defined in this way, it incorporates partitioning between the vehicle and the stratum corneum; it is an apparent, vehicle-dependent coefficient rather than a property of the barrier alone, and Kp values should only be compared between experiments using the same vehicle [15].

**Maximum flux, Jmax—µg cm^−2^ h^−1^**

The Jss obtained when the compound is applied neat or at its saturation concentration in the vehicle, that is, at maximum thermodynamic activity [15]:

(4) $$J_{max}=K_{p}\times S_{v}$$

where Sv is the saturation solubility of the compound in the applied vehicle. Jmax is therefore the upper bound of Jss attainable from that vehicle without supersaturation, and it is reported only where the donor was neat or saturated; it is not the peak flux of a finite-dose profile.

**Peak flux in finite-dose experiments, Jpeak, and its time, tpeak—µg cm^−2^ h^−1^ and h**

Under finite dosing, the donor is progressively depleted, no steady state is established, and the flux rises to a maximum that is not maintained [14,15]. The corresponding endpoint is calculated between consecutive sampling intervals:

(5) $$J_{peak}=max\left[\frac{Q_{i}-Q_{i-1}}{t_{i}-t_{i-1}}\right]$$

where Qi and Qi−1 are cumulative amounts at consecutive sampling times ti and ti−1. Jpeak should be reported together with tpeak, the time at which it occurs, since the two together characterize a finite-dose profile that has no steady-state segment. Jpeak and Jmax are distinct quantities and should not be used interchangeably.

**Lag time, tlag—h**

The intercept on the time axis of the extrapolated linear portion of the cumulative permeation profile [15]. For an ideal homogeneous membrane of thickness h and effective diffusion coefficient Deff:

(6) $$t_{lag}=\frac{h^{2}}{6D_{eff}}$$

This relation does not hold for stratified skin, in which the multilayer structure and the distribution of the compound between layers determine the observed delay. In native and reconstructed tissue, tlag should therefore be reported as an apparent, model-dependent descriptor rather than as a measure of intrinsic diffusivity [17,19].

Tlag provides complementary information on transport kinetics when a clear steady-state phase is established. The relationship between tlag, membrane thickness, and effective diffusivity for an ideal homogeneous membrane is given in Box 1, Equation (6); in native or reconstructed skin, however, the heterogeneous multilayer structure means that tlag should be interpreted as an apparent, model-dependent descriptor rather than as a direct measure of intrinsic diffusivity [17,19]. Comparative studies further show that reconstructed human epidermis can exhibit higher permeability and shorter or less pronounced tlag than human or porcine reference tissue, supporting the use of tlag as a complementary descriptor of model-dependent transport behavior rather than as an isolated measure of barrier quality [20].

Distribution endpoints describe where the drug is located after exposure. These include retention in the stratum corneum, accumulation in viable epidermis, partitioning into dermis, and transfer into the receptor phase. Such readouts are particularly important for topical products intended to act locally, where therapeutic relevance may depend more on epidermal or dermal drug levels than on total transdermal permeation. Tape stripping can provide information on stratum corneum uptake and depth distribution, whereas tissue separation or extraction can support analysis of epidermal and dermal retention [2,21,22]. Cutaneous distribution is not homogeneous within these bulk compartments: successive stratum corneum layers, distinct strata of the viable epidermis, including the stratum granulosum and stratum basale, and appendageal structures such as hair follicles and sebaceous glands can represent separate localization or reservoir domains. Consequently, bulk epidermal or dermal extraction collapses this spatial heterogeneity into a single compartment-level value, where intra-compartment localization is relevant, depth- or spatially resolved methods are required (Section 4.7) [1,21,22,23].

Metabolic endpoints are relevant when local activation, detoxification, or degradation influences efficacy or safety. These may include parent-compound depletion, metabolite formation, enzyme expression, and functional enzyme activity, with functional readouts being essential for interpretation. However, detection of enzyme mRNA or protein alone does not prove metabolic competence; compound-specific metabolite formation should be demonstrated under exposure conditions that are compatible with permeation testing. This is particularly important for ester-containing prodrugs, corticosteroid esters, cosmetic ingredients, and pro-haptens, where local biotransformation may influence retention, efficacy, irritation, or the risk of sensitization [24,25,26,27,28,29].

We use the term clearance-like removal to denote the experimental approximation of physiological clearance achieved by receptor-phase renewal or perfusion, and reserve receptor-side removal for the narrower description of what occurs at the basal boundary of the barrier. The term is deliberately broad, and for that reason, the quantities it covers should be distinguished before they are compared. The receptor-side appearance rate is the amount of compound arriving in the receptor phase or perfusate per unit time (µg h^−1^) and depends on the exposed area of the particular device. The area-normalized flux divides that rate by the exposed area (µg cm^−2^ h^−1^) and is therefore comparable between devices of different geometry, but not between compounds at different donor activities. The mass-transfer rate describes transport across a defined boundary and is the quantity actually measured at the barrier’s basal surface. Concentration-normalized clearance divides the appearance rate by the concentration in the compartment from which the compound is removed, giving units of volume per unit time, which is the only one of these quantities with the dimensions of pharmacokinetic clearance [30]. Receptor-fluid replacement, continuous flow, basal transport, and perfusate recovery are experimental means of achieving removal; they are not themselves clearance, and reporting them as such implies a physiological equivalence that the measurement does not support [31]. The corresponding expressions are given in Section 6.2, Equations (7)–(9). In static Franz diffusion cells, receptor-phase accumulation is only an experimental approximation of systemic uptake and depends on receptor medium composition, receptor volume, solubility, and sampling strategy [2,32]. Perfused and microfluidic systems can partially address this limitation by continuously renewing the receptor-side medium, improving sink-condition control, reducing unstirred-water-layer artifacts, and enabling time-resolved sampling of the perfusate [6,33,34,35]. Mass balance remains an important whole-experiment consistency check, particularly under finite-dose conditions, whereas under infinite-dose conditions, compartment-specific recovery and donor stability are often more informative than overall recovery [9,36,37,38,39,40,41,42].

Overall, pharmacokinetic endpoints should be selected based on the model’s intended use. For early formulation ranking, cumulative permeation and relative flux are often sufficient. For local dermatological products, tissue retention may be more informative than receptor-phase permeation alone. For metabolism-sensitive compounds, parent depletion, metabolite formation, and relevant functional enzyme activity should be assessed. For transdermal systems or clearance-sensitive compounds, sink conditions and dynamic clearance-like removal become central. Thus, meaningful model selection requires alignment among the pharmacokinetic question, the model’s biological capacity, and the experimental readouts collected.

### 2.2. Finite and Infinite Dose Conditions in Dermal Permeation Testing

The distinction between finite- and infinite-dose conditions is critical when comparing permeability data across in vitro skin models because it determines the donor-side boundary condition and therefore the shape and interpretation of the permeation profile.

Finite dosing is commonly used to approximate clinically relevant topical exposure, in which the amount of permeant available at the skin surface and/or its thermodynamic activity may change during the experiment due to permeation, vehicle evaporation, compositional changes, or drug crystallization. OECD TG 428 recommends application conditions that mimic human exposure, typically 1–5 mg/cm^2^ for solids and up to 10 µL/cm^2^ for liquids; these values are best regarded as practical finite-dose benchmarks rather than universal physicochemical thresholds [9,14,37].

Infinite dosing, by contrast, requires the donor compartment to remain compositionally stable over the interval used for kinetic analysis, such that permeant depletion is negligible and donor activity remains approximately constant. Provided receptor-side sink conditions are maintained, and steady state is demonstrated, cumulative permeation can approach linearity after the lag phase, allowing estimation of Jss and the Kp. This criterion applies to the vehicle as well as to the permeant: evaporation of a volatile vehicle component can change donor composition and thermodynamic activity, and may cause supersaturation or crystallization even when permeant depletion is small; infinite dosing should therefore be defined by stability of the donor boundary condition rather than by a universal volume threshold [14,15].

Operationally, dose classification should therefore be based on the observed donor and receptor boundary conditions rather than on nominal applied volume alone. An experiment may be considered demonstrably infinite over the interval used for kinetic analysis when donor depletion is measured against a predefined, system-specific acceptance criterion, donor composition remains stable without relevant evaporation, phase separation, or precipitation/crystallization, receptor-side sink conditions are verified relative to the measured solubility in the receptor medium, and a stable linear permeation interval is demonstrated. Where these conditions are only partly documented, but donor activity remains approximately constant and a linear interval is observed, the experiment is more appropriately described as quasi-infinite; material donor depletion or compositional change should be treated as finite-dose behavior. Because no universal numerical cutoff applies across compounds and vehicles, quantitative acceptance criteria should be predefined and justified for the specific experimental system.

Importantly, nominal dose classification does not always predict the observed kinetics. Hewitt et al. applied a finite dose of 10 µL/cm^2^ to 56 chemicals in an OECD TG 428-compliant human-skin protocol, yet a subset showed near-linear receptor-fluid kinetics over 24 h. For several compounds, 58–86% of the applied dose remained recoverable in the skin wash, indicating that residual material at the surface continued to act as a reservoir after solvent evaporation [36].

Finite- and infinite-dose experiments, therefore, address different pharmacokinetic questions. Infinite-dose conditions are most informative for characterizing steady-state transport and parameterizing diffusion models, whereas finite-dose experiments better reproduce the evolving donor conditions relevant to topical product use. Accordingly, Jss and Kp should be regarded as system-dependent permeability parameters rather than intrinsic properties of the skin barrier, and data generated under finite- and infinite-dose conditions should not be treated as directly interchangeable when comparing skin models or formulations [14,15].

### 2.3. Distinction Between In Vitro Release and Permeation Testing

In vitro release testing (IVRT) and IVPT address distinct aspects of topical product performance and should not be interpreted interchangeably. IVRT measures the rate of drug release from a topical formulation across a synthetic membrane into a receptor medium. The membrane is commonly a porous synthetic filtration membrane selected according to pore size, chemical compatibility, and minimal drug binding; its inertness and the linearity and precision of the resulting release kinetics should be demonstrated during method development and validation. IVRT, therefore, characterizes formulation-dependent drug release under defined experimental conditions rather than transport across a biological skin barrier [43].

A further distinction concerns the physical interpretation of the derived rate parameters. In IVRT, the synthetic membrane should be non-rate-limiting, and release is commonly evaluated from the linear relationship between cumulative amount released per unit area and the square root of time (Higuchi-type analysis), yielding a release-rate slope in µg cm^−2^ h^−1^ᐟ^2^ [43]. In IVPT, the skin itself contributes diffusional resistance; under conditions supporting steady-state transport, cumulative permeation is evaluated against time within a Fickian framework, yielding flux in µg cm^−2^ h^−1^ and, where appropriate, Kp [10]. Because these rate parameters have different physical meanings and dimensions, IVRT release rates should not be numerically equated with IVPT fluxes or interpreted as interchangeable measures of product performance.

IVPT, in contrast, characterizes the rate and extent of a drug’s permeation from a topical product through excised skin under defined conditions [10]. Receptor-phase flux is not itself a measure of target-site concentration; additional layer-specific measurements are required when local tissue availability is the endpoint of interest [2,21]. For bioequivalence-oriented IVPT, the FDA recommends using excised human skin and qualifying stratum corneum barrier integrity. The resulting flux profile reflects the combined effects of drug availability from the formulation and permeation through the skin and is evaluated using endpoints such as maximum flux (Jmax) and total cumulative amount permeated (AMT) [10]. IVPT, therefore, provides information on cutaneous drug availability that cannot be inferred from IVRT alone [10,43].

The two methods are consequently complementary rather than interchangeable within regulatory assessment, physicochemical and structural (Q3) characterization describes physicochemical and structural attributes of the formulation that may influence product performance; IVRT compares drug-release performance, whereas IVPT compares the rate and extent of drug availability through the skin. An inert filtration membrane suitable for IVRT should therefore not be interpreted as a surrogate for the skin barrier in IVPT [10,43,44]. Skin-mimetic synthetic membranes such as Strat-M^®^ serve a different experimental purpose: they are designed to reproduce selected structural and chemical features of the stratum corneum and have been used as standardized barriers in research permeation studies, but they lack the viable biological components of native skin [45,46,47,48].

### 2.4. Mass Balance, Recovery Criteria, and Analytical Constraints

Mass balance is most informative in finite-dose experiments, in which a defined applied dose should be recoverable from the surface wash, residual donor, stratum corneum, viable epidermis and dermis, receptor phase or perfusate, and apparatus. In infinite-dose experiments, overall recovery is dominated by the residual donor and is therefore less informative for assessing low-abundance tissue or receptor compartments; donor stability, receptor-side sink conditions, and compartment-specific analytical recovery should be evaluated separately. When recovery is incomplete, a low permeation value cannot be distinguished from analytical loss [14,36,38]. Conversely, acceptable overall recovery does not establish the accuracy of a low-abundance compartment, because a loss that is small relative to the applied dose may be large relative to the amount measured in that compartment. Overall mass balance should therefore be interpreted as a whole-experiment consistency check rather than as evidence of compartment-specific analytical accuracy.

Regulatory expectations are explicit and should be stated numerically. OECD TG 428 defines acceptable mean recovery as 90–110% for radiolabeled test substances [9]; Guidance Document 28 retains these limits and extends them to approximately 80–120% for volatile or non-radiolabeled substances [37]; Guidance Document 156 sets out how absorption values should be normalized, or treated conservatively by assigning unrecovered material to the absorbed fraction, when these limits are not met [11]. The EMA guideline applies the same 90–110% window to overall recovery in IVPT [12]. These limits are frequently not met even under standardized conditions: approximately one-third of mass balance values in the European Food Safety Authority (EFSA) dermal absorption database fall outside EFSA’s narrower 95–105% criterion [39], 48% of 50 chemicals tested by the Cosmetics Europe ADME Task Force fell below 90%, predominantly through evaporation [40], and within a single 56-chemical study, recovery ranged from 20% for a volatile compound to 97% in the same experiment [36]. Kluxen et al. further argue that unrecovered material represents random negative deviation from the nominal dose, and that generic application of the addition rule inflates rather than corrects the absorption estimate [39]. Recovery should therefore be reported rather than assumed, and both the acceptance criterion and the denominator used for its calculation should be stated explicitly.

Incomplete recovery should also be investigated according to its likely mechanism rather than treated as an undifferentiated missing fraction. A practical stepwise assessment is to verify the actually delivered dose, then evaluate physical losses through volatilization or adsorption to the apparatus, validate extraction recovery and chemical stability in the relevant matrices, and, in metabolically competent systems, assess whether unmeasured metabolites account for the deficit. Percentage recovery should be calculated against a clearly stated denominator; where feasible, a gravimetrically or analytically verified delivered dose is preferable to the nominal target dose, because application error would otherwise be confounded with experimental loss.

Analytical constraints interact directly with model choice. Reconstructed and bioprinted constructs expose small surface areas and small tissue masses, so the amount available for quantification in the epidermal or dermal compartment may approach the limit of quantification even when the permeation experiment is sound, and extraction efficiency from a collagen, fibrin, or gelatin methacryloyl (GelMA) matrix requires separate validation for each construct rather than assumed equivalence to native dermis. In perfused and microfluidic platforms, absorption of lipophilic compounds into bulk poly(dimethylsiloxane) (PDMS) channels and tubing can remove a substantial fraction of the applied dose from the measurable compartments, simultaneously depressing apparent flux and breaking mass balance; the extent of loss depends on device geometry, exposure time, and permeant properties, and is large enough to alter measured drug responses [41,42]. Recovery is therefore an important whole-experiment consistency check, but it does not substitute for compartment-specific analytical validation.

### 2.5. Barrier Integrity Testing: Methods and Acceptance Criteria

Every quantitative statement in this review presupposes that the barrier was intact at the start of the experiment. Barrier integrity testing is required by OECD TG 428 and by the FDA and EMA guidance on IVPT, which additionally requires that the choice of test and its acceptance criterion be explained and justified, yet it is frequently reported only as having been performed, without the method or the threshold applied [9,10,11,12,37]. Because an undetected defect is indistinguishable from enhanced permeation, the acceptance criterion should be considered part of the experimental context in which permeation data are interpreted.

Three methods are established for this purpose: transepidermal water loss (TEWL), electrical resistance (ER), and tritiated water permeability. Their measurands, reported criteria, and limitations are collected in Supplementary Table S1, along with two complementary approaches (lipophilic probe permeability and visual or histological inspection) that are not yet established as stand-alone acceptance tests. ER has been shown to be an appropriate substitute for the tritiated water Kp in identifying intact membranes [49,50], although agreement between integrity measurements on individual skin samples and the absorption of specific test compounds is considerably weaker [51]. Three points govern interpretation. First, none of the three tests has a harmonized threshold: tritiated water criteria span a threefold range across otherwise comparable regulatory studies, and TEWL criteria range from a one-sided limit to a two-sided window set based on laboratory history rather than validated against transport data [36,49,50,51,52]. Second, ER criteria are meaningful only when normalized to the exposed area, and a retrospective analysis of 9978 measurements found that the widely used 10 kΩ limit for a 2.54 cm^2^ cell flagged approximately half of all samples, many of them false positives, with 5 kΩ performing better without affecting the measured absorption [52]. Third, passing any criterion establishes only that tissue is not grossly damaged, not that barrier quality is comparable among samples that pass: dermal delivery across 56 chemicals correlated neither with applied dose nor with TEWL values within the accepted range [36], and agreement between tritiated water and a lipophilic probe such as radiolabeled octanol is poor, so a pass on a water-based test does not guarantee that lipophilic transport is unaffected [53].

These observations distinguish four related but non-equivalent concepts. Barrier integrity is an exclusion-oriented assessment of gross damage; functional barrier performance is the quantitative transport behavior of a section for a given permeant; barrier comparability concerns differences among sections that all pass the integrity criterion; and barrier maturation in reconstructed or bioprinted constructs refers to the degree of differentiation and lipid organization achieved at use [5,54]. Integrity testing in ex vivo skin is therefore primarily a prospective exclusion tool and does not establish equivalent barrier performance among accepted sections. Where a non-destructive integrity measure is obtained before dosing, the measured values should also be reported for accepted sections and may, if prespecified, support balanced treatment allocation or stratification and sensitivity analyses of baseline barrier variation. Reconstructed and bioprinted constructs require separate assessment of maturation and barrier quality, whereas synthetic membranes require controls for material integrity and vehicle compatibility rather than biological integrity testing [55].

### 2.6. Variability, Replication, and Statistical Design in IVPT

IVPT data generated with ex vivo human skin have a hierarchical structure because several skin sections are commonly obtained from the same donor. Replicate sections from one donor, therefore, characterize within-donor variability, but should not be regarded as independent observations of population-level skin variability. This distinction is biologically relevant: in standardized Franz-cell experiments with caffeine, methylparaben, and butylparaben, intra-donor variability in permeability was generally lower than inter-donor variability, although the magnitude of the difference was compound-dependent. Akomeah et al. further found greater variability for the more hydrophilic caffeine than for the parabens, illustrating that variance itself can depend on permeant properties [56].

Study design should therefore preserve donor identity and exploit within-donor comparisons rather than pool all diffusion cells as independent replicates. The FDA draft IVPT guidance recommends that test and reference products in pivotal studies should be evaluated on skin from the same donors, with equal numbers of replicate sections per treatment, and that individual sections be randomly assigned to treatment. For method-validation pilot studies, the draft guidance recommends approximately 4–6 donors, with at least 4 replicate skin sections per donor per treatment group; for pivotal studies, the number of donors should be justified by statistical power, while at least 4 dosed replicates per donor per treatment are recommended. Results should be retained at the individual diffusion-cell level and summarized separately for intra-donor and inter-donor variability rather than reported only as an overall mean. The general methodological lesson is therefore fourfold: a donor is not a replicate; donor identity must be preserved; comparisons should be matched within donor wherever possible; and both within- and between-donor variance should be modeled and reported [10].

Statistical dependence also extends within each diffusion cell. Repeated receptor samples share the same donor, barrier, dose, and receptor compartment, and cumulative permeation values are, by construction, correlated because each value includes the preceding amounts. Accordingly, analysis of complete permeation profiles should account for repeated measurements within cells, for example, using mixed-effects or repeated-measures models with donor and diffusion cell nested within donor as random effects, treatment, time, and their interaction as fixed effects, and treatment-by-donor variability represented where appropriate. By contrast, analyses of cell-level-derived endpoints, such as Jss, maximum or peak flux, or total amount permeated, use a single summary value per cell and should retain the donor/replicate hierarchy. These approaches answer different questions and should be reported separately. Recent comparative analyses likewise show that IVPT bioequivalence methods must accommodate within-donor replication and treatment–donor variability and that model-based results depend on correct variance specification [57,58].

### 2.7. Common Methodological Sources of Bias in Skin Permeation Studies

Methodological differences can mimic, amplify, or obscure genuine differences between skin models. Importantly, these effects are not always random or uniformly directional. Loss of receptor-side sink conditions, excessive receptor boundary-layer resistance, undetected barrier damage, and compound sorption to experimental materials have mechanistically predictable effects on measured permeation, whereas storage effects, formulation metamorphosis, and membrane–vehicle interactions can vary with the permeant, vehicle, and experimental protocol [32,33,42,59]. Meaningful cross-study comparison, therefore, requires transparent reporting of tissue or model provenance, dosing and receptor conditions, barrier-integrity assessment and its acceptance criterion, replicate and donor structure, and analytical and mass balance recovery. Table 1 summarizes the principal recurrent sources of bias and their expected effects on pharmacokinetic readouts; the indicated directions are intended as an interpretative aid rather than as correction factors.

Collectively, these methodological considerations define the experimental context within which model-dependent pharmacokinetic differences can be interpreted. Differences in flux, permeability, tlag, or tissue retention cannot be attributed confidently to the skin model itself unless conditions, receptor-side transport, barrier integrity, donor or batch structure, and analytical recovery are adequately controlled and reported. Consequently, meaningful comparison of in vitro skin models requires consideration of both their biological capabilities and the experimental boundary conditions under which their pharmacokinetic outputs are generated.

## 3. Absorption: Model-Dependent Prediction of Skin Permeation

Absorption data generated with in vitro skin models should be interpreted as model-specific outputs rather than interchangeable estimates of human skin permeation. Differences in barrier architecture, tissue compartments, dose conditions, and receptor-side boundary conditions can alter both the magnitude and rank order of permeation across model classes.

### 3.1. Synthetic Membranes

Synthetic membranes provide highly reproducible physicochemical barriers for early absorption screening, but their biological interpretation is limited. Strat-M^®^ is among the best-characterized synthetic barriers used in comparative skin-permeation studies. It contains a layered structure with a tight upper layer and lipid components intended to approximate selected features of stratum corneum chemistry, but it lacks viable cells, native lipid organization, appendages, metabolism, and biologically meaningful tissue retention [45]. Its principal value is therefore in reproducible formulation ranking and comparative permeation screening rather than in reproducing the complete transport behavior of native human skin.

Uchida et al. compared Strat-M^®^ with excised human skin and hairless rat skin using 13 compounds with log P values from −0.9 to 3.5 and reported correlations in Kp across this range, supporting its use for comparative screening across chemically diverse compounds [47]. Haq et al. similarly reported good correlation between nicotine permeation through Strat-M^®^ and human cadaver skin, including enhancer-containing formulations [45], while studies of rivastigmine transdermal systems and amphotericin B nanoformulations have reported strong correlations or similar permeation profiles under defined experimental conditions [48,61]. Together, these findings support the use of Strat-M^®^ for comparative screening across diverse permeants and formulations, while correlation with native skin should not be interpreted as quantitative agreement or equivalence in absolute flux.

Direct head-to-head comparisons, however, show that the magnitude and direction of differences from biological skin are not constant. Kichou et al. reported resorcinol Jss of 40–42 µg/cm^2^/h across Strat-M^®^, 138–162 µg/cm^2^/h across EpiSkin^®^, and 5–13 µg/cm^2^/h across excised human skin [46]. Huh et al. likewise reported substantially higher permeability of 1,2-benzisothiazolin-3-one through Strat-M^®^ than through minipig skin under infinite-dose conditions [62]. Taken together, these data indicate that synthetic-to-native differences are compound-, vehicle-, and dose-dependent and do not currently support a single universal correction factor.

Dose and vehicle conditions are central to this interpretation. Arce et al. showed that Strat-M^®^ can be useful for assessing permeation from complex vehicles under finite-dose conditions, whereas predictivity decreased under infinite-dose conditions and in formulations with high polyol content, where membrane integrity may be affected [55]. These findings further emphasize that synthetic membrane performance is contingent on the donor regimen and membrane–vehicle compatibility, rather than being an intrinsic property of the membrane alone. A mechanistic limitation follows from the absence of native lipid organization. Chemical penetration enhancers acting by fluidizing, disrupting, or extracting intercellular stratum corneum lipids have no equivalent substrate in Strat-M^®^, so any effect observed there reflects vehicle-side changes in solubility or partitioning rather than the mechanism operating in native skin. Agreement reported for enhancer-containing formulations [45] therefore cannot be generalized, and the enhancement attributed to lipid perturbation should be confirmed in a tissue-based model.

Silicone membranes, including PDMS-based barriers, are simpler synthetic systems than Strat-M^®^. They are hydrophobic, chemically stable, and readily integrated into diffusion or microfluidic platforms, but lack stratum-corneum-like lipid architecture. They are therefore used principally as reproducible passive-diffusion barriers and for device or platform validation [33]. However, sorption of lipophilic compounds to PDMS can alter effective exposure, analytical recovery, and apparent transport kinetics [63].

Overall, synthetic membranes are best positioned as reproducible tools for formulation screening and comparative permeation rather than as universal surrogates for human skin. Strat-M^®^ has one of the broadest comparative evidence bases among synthetic skin-mimetic membranes, but its predictive performance remains compound-, formulation-, and protocol-dependent. Questions requiring native barrier architecture, biologically meaningful tissue partitioning, or human-reference permeation are therefore better addressed using ex vivo skin or an appropriately qualified biological model.

### 3.2. Ex Vivo Human Skin

EHS remains the most physiologically relevant conventional reference model for in vitro skin absorption studies because it preserves the native multilayer architecture of human skin, including the stratum corneum, viable epidermis, and dermis [1,2,54]. EHS is an established tissue source for in vitro dermal absorption testing and serves as a principal comparator when evaluating alternative human cell-based models [8,9,20,64]. Compared with synthetic membranes and epidermis-only reconstructed models, EHS better captures the native lipid organization, corneocyte architecture, tissue thickness, appendageal pathways, and dermal reservoir capacity that determine permeability, flux, and tissue partitioning [5,45,46,54,64,65].

In Franz-cell IVPT, EHS supports measurement of cumulative receptor-phase permeation, Jss, Kp, tlag, and drug recovery across the donor, skin surface, and tissue compartments. These readouts support formulation comparison, bioequivalence-oriented IVPT [2,10,12,13], and the generation of experimental inputs for IVIVE or systemic exposure modeling [66,67,68,69,70]. Unlike synthetic membranes, EHS additionally preserves biologically relevant partitioning into viable skin compartments.

A major limitation of EHS is biological and preparative heterogeneity. EHS should not be treated as a single experimental preparation: full-thickness skin, dermatomed skin, and isolated epidermal membranes differ in viable-tissue content, dermal reservoir capacity, and transport resistance. Akomeah et al. provided an important benchmark for human skin variability by compiling caffeine Kp from multiple anonymous donors using heat-separated human epidermis in standard Franz cells. Reported Kp values ranged from 0.5 to 2.5 × 10^−3^ cm/h, with intra-subject variability generally lower than inter-subject variability. These data illustrate substantial inter-donor variability and, for a hydrophilic permeant such as caffeine, the sensitivity of measured permeability to differences in barrier properties [56]. Therefore, permeability values obtained with EHS should be interpreted alongside information on tissue preparation, donor, anatomical site, storage history, and barrier integrity.

The difficulty of replacing EHS with a single alternative model is illustrated by direct comparative studies. Salminen et al. tested EHS, EpiDerm-200-X, (MatTek Life Sciences, Ashland, MA, USA), and Strat-M^®^ (Millipore, Merck KGaA, Darmstadt, Germany) under identical finite-dose conditions using caffeine, salicylic acid, and testosterone. The 6 h cumulative permeation ranking was compound-dependent: caffeine and testosterone permeated most through EpiDerm-200-X, followed by EHS, and then Strat-M^®^, whereas salicylic acid permeated most through EHS [64]. This matched comparison demonstrates that alternative models do not exhibit a uniform directional bias relative to EHS; model ranking can change with the permeant and experimental conditions.

As discussed in Section 2.2, the interpretation of EHS permeation depends strongly on the donor boundary condition. Finite-dose designs can better reproduce the evolving donor conditions associated with topical application, whereas infinite-dose experiments address different mechanistic or steady-state transport questions; data generated under the two conditions should therefore not be treated as directly interchangeable [2,13,55].

Because EHS retains native skin compartments, receptor-phase permeation can be interpreted together with tissue retention and depot formation. Layer-specific distribution is considered separately in Section 4. EHS nevertheless lacks active blood and lymphatic flow, so receptor-phase appearance should not be interpreted as physiological vascular clearance.

Storage and tissue handling are major limitations of EHS. Barrier performance can be affected by tissue preparation, storage duration, and storage conditions, all of which should be documented when interpreting EHS permeation data [2]. The most direct experimental characterization of freeze–thaw damage to the stratum corneum has been performed in animal tissue and is therefore considered in Section 3.3. Accordingly, the magnitude of freeze–thaw effects observed in animal skin should not be extrapolated quantitatively to EHS without human-specific evidence.

Because biological variability is intrinsic to EHS, rigorous control of tissue preparation, receptor conditions, barrier integrity, donor-level replication, and analytical recovery is essential for interpretable comparisons; these methodological determinants are discussed in detail in Section 2 [2,9,13].

Overall, EHS remains the principal conventional reference model for human-relevant absorption because it combines native barrier architecture with measurable receptor-phase permeation and tissue retention. Its main limitations are limited availability, donor- and anatomical-site variability, preparation- and storage-sensitivity, and the absence of active vascular clearance. These limitations motivate the use of alternative models, but do not eliminate the need for EHS as a human-reference comparator when quantitative human relevance is required.

### 3.3. Ex Vivo Animal Skin

Ex vivo animal skin is widely used as an alternative or comparator to excised human skin in IVPT because it is more accessible, less expensive, and less limited by donor availability. It is particularly useful in early formulation screening, penetration-enhancer studies, and comparative IVPT experiments, where the aim is to rank formulations or estimate relative absorption rather than to reproduce human skin exactly [2,4]. However, animal skin should not be treated as a single model category. Pig, rat, mouse, rabbit, and guinea pig skin differ in stratum corneum thickness, lipid organization, follicular density, and viable-layer thickness, all of which can influence permeability, flux, and tissue retention [4,71,72]. Part of this difference is anatomical rather than species-related. Ex vivo human skin is usually obtained from the abdomen or breast, whereas the commonest comparators, porcine ear skin in particular, carry a much higher follicular density, which varies markedly both between anatomical sites and between species [73,74]. Because follicles provide an additional penetration and reservoir pathway, apparent species differences partly reflect the donor sites used, and the anatomical site should therefore be reported alongside the species.

Among animal models, porcine skin is generally considered one of the most useful practical surrogates for human skin absorption. It is anatomically and functionally closer to human skin than most laboratory rodent models, particularly in epidermal thickness, dermal organization, hair-follicle pattern, and barrier function [4,71]. In the quantitative review by Barbero and Frasch, pig and human skin permeability showed a strong correlation, supporting porcine skin as one of the most human-relevant practical animal comparators for IVPT studies [4].

Practical IVPT studies further support this role. Brighenti et al. tested benzoyl peroxide retention and permeation profiles of marketed topical formulations on porcine and human skin under identical conditions and reported comparable rank orders and quantitative ranges. This matched comparison supports the use of porcine skin for relative formulation discrimination, while not establishing quantitative equivalence with human skin [75].

Nevertheless, porcine skin is not interchangeable with human tissue. Kocsis et al. compared human, pig, mouse, and rat skin and showed that some basic biophysical parameters, such as hydration and pH, may be similar across species, while freezing, aging, and tissue handling can alter barrier function. They also demonstrated species-dependent differences in skin thickness and biochemical composition, reinforcing that similarity in selected surface parameters does not imply equivalent barrier behavior [72]. Accordingly, species selection should follow the research question: porcine skin is generally the most human-relevant practical animal alternative when EHS is unavailable, particularly for comparative permeation studies and formulation rank ordering, whereas rodent skin may be useful for controlled mechanistic or preclinical comparisons but is less suitable for quantitative prediction of human flux [4,71,72,75,76].

Storage and tissue handling are further sources of variability. In pig ear skin, Abdayem et al. showed that freezing and thawing disrupted stratum corneum ultrastructure and tight-junction function, with a corresponding increase in the Kp of a hydrophilic permeant relative to fresh tissue [77]. Consistent with this, altered caffeine permeation has also been reported in frozen/thawed animal skin used for formulation studies [78]. These findings show that storage history can become a major experimental determinant, particularly for permeants sensitive to disruption of polar barrier pathways; freeze–thaw effects should therefore be reported and interpreted as tissue- and permeant-dependent rather than assumed to be negligible [77,78].

A major limitation of several animal models is their tendency to exhibit greater permeability than human skin, although the magnitude and direction of the difference are species- and compound-dependent. Jung and Maibach concluded that monkey, pig, and hairless guinea pig are generally more predictive of human absorption, whereas common laboratory animals such as rat, rabbit, and other guinea pig preparations tend to overestimate human skin absorption or penetration [71]. Higher animal-skin permeability, therefore, does not justify treating animal flux as quantitatively equivalent to human absorption [4,71].

The human relevance of animal skin is also compound-dependent. Simon K. et al. [76] tested the dermal penetration of 24 polycyclic aromatic hydrocarbons across human and porcine skin in Franz diffusion cells and found human skin to be less permeable than pig skin. More lipophilic PAHs showed lower permeation into the viable epidermis and dermis, while large PAHs with log P ≥ 6 did not fully permeate pig skin even after 48 h. These findings further demonstrate that human–porcine differences depend on permeant properties and that even porcine skin cannot be assigned a fixed scaling relationship to human absorption [76].

Because animal skin retains viable tissue compartments, permeation can be interpreted together with tissue retention, although species differences limit direct extrapolation of compartment-specific exposure to humans. Layer-specific distribution is considered in Section 4. As with EHS, receptor-phase appearance represents transfer into an experimental sink rather than physiological vascular clearance.

Overall, ex vivo animal skin occupies an intermediate position between synthetic membranes and excised human skin. It provides greater biological complexity than Strat-M^®^ or PDMS by retaining native skin layers, lipid domains, appendages, and tissue binding sites, but remains less directly human-relevant than EHS. Among animal models, porcine skin is generally the most informative practical comparator for comparative IVPT and formulation ranking, whereas other laboratory animal models often show greater divergence from human permeability [4,71]. Importantly, even human–porcine relationships remain compound and protocol-dependent and do not currently support a single universal scaling factor [76]. Animal skin, therefore, remains valuable for comparative and mechanistic studies, while reconstructed human skin models offer an attractive alternative when greater standardization and human cellular origin are priorities.

### 3.4. Reconstructed Human Epidermis and Full–Thickness Skin Equivalents

RHE, living skin equivalents (LSE), and FTSE represent cell-based human skin models developed to reduce reliance on excised human and animal skin. RHE models consist primarily of keratinocytes organized into a stratified epidermis with a stratum corneum-like surface, whereas LSE/FTSE constructs additionally include a dermal compartment, typically composed of fibroblasts embedded in collagen or other extracellular matrix scaffolds [5,20,65]. Their main advantage in absorption studies is standardization: compared with excised human skin, these models are produced under more controlled and less donor-dependent conditions and are readily compatible with standardized Franz-cell protocols, although batch- and supplier-related variability remains [8,20,79].

The absorption behavior of commercial RHE models was systematically evaluated in the Schäfer-Korting prevalidation and validation studies. In the three-laboratory prevalidation study, EPISKIN^®^, EpiDerm^®^, and SkinEthic^®^ were compared with ex vivo bovine udder, pig, and human epidermis in response to testosterone and caffeine. For both permeants, reconstructed models generally showed higher permeability than the native reference preparations, although the magnitude of the difference varied among models. The study also demonstrated inter-laboratory reproducibility and preservation of useful rank-order information [20]. The subsequent formal validation study expanded the test panel to nine substances across ten laboratories and confirmed the overall pattern: RHE frequently exhibited higher permeability than human epidermis or pig skin, while retaining useful substance rank-order correspondence with the reference tissues under finite- and infinite-dose conditions [8]. Together, these programs support RHE as a reproducible comparative platform, but not as a quantitatively interchangeable substitute for native human skin.

The higher permeability frequently observed in RHE is mechanistically linked to incomplete maturation of the stratum corneum barrier. Human skin equivalents do not fully reproduce the barrier function of native human skin, largely because their stratum corneum lipid and protein organization remains non-native [5]. Reduced free fatty acid content, altered ceramide subclass distribution, and a shift from native orthorhombic lipid packing toward less ordered lipid phases can create more permissive diffusion pathways [5,54]. These structural differences are also reflected in kinetic readouts: RHE tends to exhibit shorter tlag and higher Jss than human or pig skin, and the Schäfer-Korting prevalidation study specifically reported that the long and variable tlag observed with native tissues were absent in RHE [20]. Thus, shorter, less variable tlag may facilitate comparative screening but may underrepresent the transport delay imposed by the native barrier. Importantly, these structural differences provide a mechanistic basis for the higher permeability observed in many RHE studies without implying a constant or compound-independent bias.

RHE can accommodate finite-dose application and the associated evolution of donor conditions at the surface, but it cannot fully reproduce the behavior of the native tissue reservoir. Because epidermis-only RHE lacks a dermal compartment and differs from native stratum corneum in barrier organization, deeper tissue retention and dermal reservoir effects cannot be represented. This limits its interpretation when receptor-phase kinetics are strongly influenced by tissue partitioning or prolonged retention [20,79].

The addition of a dermal compartment in LSE/FTSE expands the interpretation of absorption by allowing dermal partitioning and retention to influence receptor-phase appearance. FTSE constructs provide a greater diffusion distance and additional binding capacity within the dermal equivalent, potentially reducing apparent flux and increasing tissue retention compared with RHE alone. Ackermann et al. tested the Phenion^®^ full-thickness skin model in Franz-type diffusion cells and found that it was more permeable than pig skin, but that its dermal compartment created an additional reservoir, particularly for the lipophilic compound testosterone [65]. This demonstrates that the addition of a dermal equivalent can modify receptor-phase kinetics through tissue partitioning and retention, although the Phenion^®^ model remained more permeable than pig skin. Thus, adding structural complexity expands the range of pharmacokinetic processes that can be represented but does not, by itself, establish native-skin equivalence.

Direct comparative data further demonstrate that reconstructed-skin performance is compound and model-dependent. Permeation through EpiDerm-200X was higher than through excised human skin and Strat-M^®^ for caffeine and testosterone under matched finite-dose conditions, whereas for salicylic acid, the rank order reversed, with human skin showing the highest permeability [64]. This matched comparison demonstrates that the deviation of RHE from ex vivo human skin is neither uniform in magnitude nor necessarily consistent in direction, so that the available data do not support a simple universal correction or scaling factor.

RHE may therefore be particularly useful for bioequivalence-style comparative testing, where discriminatory performance under matched conditions may be more relevant than reproduction of absolute human permeability. Agonia et al. used RHE in a modified IVPT approach to distinguish between clotrimazole creams of different strengths without placebo interference [79]. This illustrates how a model with imperfect absolute predictive power for humans may nevertheless provide useful comparative product discrimination.

Overall, RHE and LSE/FTSE occupy an intermediate position between ex vivo skin and more advanced vascularized or perfused platforms. Their principal value lies in standardized, human-cell-based comparative testing, with LSE/FTSE extending the experimentally accessible space to include dermal partitioning and retention. However, non-native barrier organization and, depending on the construct, the absence of appendages and physiological vascular removal limit the uncalibrated prediction of absolute human permeability [5,8,20,64,65]. These models should therefore be regarded primarily as fit-for-purpose comparative platforms; quantitative translation requires model-specific benchmarking or calibration against an appropriate human reference.

### 3.5. Advanced Engineered Full-Thickness Constructs: Vascularized and Bioprinted Systems

Vascularized and bioprinted full-thickness constructs are among the most structurally customizable groups of advanced in vitro skin models, but the terms describe different aspects of model design and should not be used interchangeably: bioprinting refers to the fabrication method, whereas vascularization refers to the presence of vascular-like structures and may be achieved with or without printing. Bioprinting deposits cells and matrix in spatially defined patterns and can therefore provide spatial control over cellular and extracellular-matrix organization [7,80,81]. Vascularized full-thickness equivalents may instead be produced by molding, self-assembly, and microfluidic integration without a printing step [7]. Depending on construct design, these platforms can extend absorption studies beyond epidermal transport to include dermal partitioning, appendage-associated pathways, and, when perfused, dynamic removal from the dermal side.

One of the clearest pharmacokinetic examples to date is a perfusable vascularized full-thickness skin equivalent produced by molding, self-assembly, and microfluidics rather than by bioprinting. The construct contained a differentiated epidermis and perfusable vascular channels connected to a self-assembled microvascular network [7]. Caffeine and minoxidil were applied topically to assess transport from the surface toward the vascularized dermal compartment, whereas benzo[a]pyrene was delivered through the perfusion circuit to evaluate vascular-to-tissue transport. This bidirectional design enabled both topical-to-vascular and vascular-to-skin transport within the same construct [7].

For absorption modeling, the principal added value of vascularized or perfused constructs is therefore their ability to couple epidermal penetration with dynamic removal from the dermal side rather than structural complexity itself. This may be particularly relevant when receptor-phase kinetics are influenced by dermal retention or downstream transport; the underlying receptor-side mechanisms are examined in Section 6. However, predictive absorption performance remains critically dependent on the epidermal barrier’s maturity. As discussed in Section 3.4 for reconstructed models, non-native stratum corneum lipid and protein organization can produce permeability that differs substantially from EHS [5,54]. Vascularization or perfusion can therefore expand the range of pharmacokinetic processes that can be represented, but cannot compensate for an inadequately characterized epidermal barrier.

Bioprinting additionally provides experimental control over dermal geometry, cellular organization, and matrix composition, although evidence that these capabilities translate into quantitatively improved absorption prediction remains limited. Maggiotto et al. demonstrated prolonged perfused culture of a bioprinted vascularized skin-on-chip using GelMA-based layers and sacrificial Pluronic F127 channels, establishing the feasibility of continuous perfusion within a printed construct, although quantitative drug-permeation endpoints were not the primary focus of the study [80]. Quantitative permeation evidence from genuinely bioprinted skin remains sparse, and standardized comparisons of Kp, Jss, and tlag against ex vivo human skin across chemically diverse compounds are still lacking [2,7]. Robust comparative benchmarking against reference tissue will therefore be required before quantitative predictive claims can be established.

A further absorption-relevant capability is the incorporation of appendage-like structures. Motter Catarino et al. bioprinted dermal-papilla-cell and HUVEC spheroids within a fibroblast-containing dermal layer and demonstrated maturation into hair-follicle-like structures supported by keratinocyte and melanocyte migration [81]. Because native hair follicles provide appendage-associated penetration and reservoir pathways [23], such constructs may ultimately enable the investigation of transfollicular delivery. However, the Motter Catarino study demonstrates architectural incorporation of follicle-like structures rather than validated transfollicular drug transport.

Overall, vascularized and bioprinted full-thickness constructs are currently best positioned as mechanistic platforms that expand the range of pharmacokinetic processes accessible in vitro, rather than as established quantitative substitutes for human-reference IVPT. Their value lies in customizable human-cell-based architectures that can incorporate epidermal, dermal, vascular, and appendage-like compartments, while their quantitative predictive performance remains constrained by epidermal barrier maturity and limited benchmarking and inter-laboratory validation [2,5,7,80,81]. Thus, architectural capability should be distinguished from demonstrated predictive validity. Because vascularization, bioprinting, and microfluidic perfusion can coexist within the same platform, these constructs overlap substantially with the skin-on-chip systems discussed in Section 3.6.

### 3.6. Perfused and Microfluidic Skin-on-Chip Systems

Perfused and microfluidic skin-on-chip systems provide a dynamic alternative to conventional static diffusion-cell formats. Their defining feature is the replacement of a static receptor phase with continuous or semi-continuous flow beneath the membrane or tissue. Continuous receptor renewal reduces downstream accumulation and can provide more stable receptor-side boundary conditions than a static chamber, although it remains an experimental approximation rather than physiological dermal clearance [2,33,34]. This distinction is particularly relevant when solute accumulation or boundary-layer resistance in static diffusion cells becomes rate-limiting, thereby influencing the apparent permeation profile [2,33]. The mechanistic consequences of sink control and receptor-side resistance are considered further in Section 6.

Experimental comparisons demonstrate that microfluidic flow can improve receptor-side control and kinetic resolution. Alberti et al. developed a multi-chamber microfluidic platform for skin permeation testing and compared it with the Franz cell using caffeine, salicylic acid, and testosterone. With silicone membranes, the microfluidic chip provided higher sensitivity in cumulative permeated amounts and comparable or lower coefficients of variation than the Franz cell. Using an organotypic skin culture, the same platform reduced unstirred-water-layer effects observed in static Franz cells, supporting the value of flow when receptor-side transport becomes rate-limiting [33].

Pulsoni et al. directly compared a conventional Franz diffusion cell with the multi-in-vitro-organ (MIVO) microphysiological system using Strat-M^®^ and pig ear skin under standardized conditions. Both systems showed similar trends for caffeine and the lipophilic compound LIP1, whereas MIVO showed closer agreement with the selected reference behavior for LIP1, which the authors attributed to more physiological fluid flow below the skin model [6]. This comparison supports the potential value of dynamic receptor flow when downstream transport contributes materially to the measured permeation profile, while also indicating that its effect may be compound-dependent.

Tárnoki-Zách et al. further demonstrated that a three-layer human skin equivalent could be integrated into a dynamic microfluidic diffusion chamber and generate caffeine transport kinetics broadly comparable with excised human skin [34]. This finding demonstrates the compatibility of reconstructed barriers with dynamic platforms but does not, by itself, establish that microfluidic flow improves the biological validity of the tissue construct.

For absorption studies, the principal strength of microfluidic platforms lies in their methodology rather than in their inherent biology. Continuous receptor renewal and frequent effluent sampling can improve temporal resolution, maintain more controlled receptor-side conditions, and reduce the risk that downstream accumulation obscures barrier-controlled transport [2,6,33]. However, the biological relevance and absolute permeability of the resulting system remain primarily determined by the barrier integrated into the platform, whether Strat-M^®^, pig or human skin, RHE, FTSE, or a custom skin equivalent [6,33,34]. Dynamic flow, therefore, expands control over receptor-side transport but does not compensate for limitations of the biological barrier itself.

Overall, perfused and microfluidic skin-on-chip systems are particularly useful when receptor-side conditions materially influence permeation kinetics. They enable continuous receptor renewal, improved sink control, and time-resolved sampling, and may reduce tissue and formulation requirements compared with some conventional workflows [6,33,34]. Their performance nevertheless remains sensitive to device geometry, flow rate, and material sorption, while inter-laboratory standardization and endpoint-specific qualification remain limited [2,6,33,34]. These systems should therefore be viewed as complementary to Franz-cell IVPT, with their principal pharmacokinetic advantage lying in controlled dynamic receptor-side transport rather than in inherently greater biological or predictive validity.

### 3.7. Cutaneous Transporters and the Limits of a Purely Diffusive Interpretation

Most conventional skin-permeation experiments are interpreted primarily within a passive-diffusion framework. Although this remains a useful operational approximation, viable human skin also expresses xenobiotic transporters that can modify the disposition of selected compounds. ABCB1 (P-glycoprotein, P-gp), ABCC1 and ABCC2 (MRP1 and MRP2), and ABCG2 (BCRP) transcripts have been detected in human skin, with ABCC1 showing particularly prominent expression. Importantly, evidence extends beyond gene expression: MRP1 protein has been localized predominantly to hair follicles, sweat glands, and muscle, with moderate expression in the basal epidermis, and pharmacological inhibition of MRP1 reduced the cutaneous absorption of several model substrates, providing evidence of functional transporter activity in human skin. Human epidermal keratinocytes also express members of the organic anion-transporting polypeptide family, for which inhibitor-sensitive substrate uptake has been demonstrated. Transporter expression is spatially heterogeneous; P-gp, for example, is expressed predominantly in dermal structures, with weaker expression in isolated epidermis and keratinocytes [82,83,84]. Thus, both transporter identity and tissue localization are relevant when interpreting their potential contribution to cutaneous disposition.

The functional consequences of this expression profile cannot be inferred directly from transporter roles in other drug-disposition organs. Transporters conventionally classified as efflux systems may contribute to inward cutaneous disposition in a substrate- and compartment-dependent manner: inhibition of MRP1 reduced the skin absorption of several model substrates [82], while P-gp has been shown to contribute to the transdermal absorption of selected corticosteroids [84,85]. These observations indicate that transporter function depends on tissue localization, orientation, and substrate specificity. This distinction is particularly relevant when interpreting coupled skin–liver systems, in which transporter-mediated disposition in the skin compartment should be considered independently of hepatic transporter behavior.

The pharmacokinetic contribution of cutaneous transporters appears to be compound- and compartment-dependent rather than a general determinant of dermal absorption. In Mdr1a/1b/Bcrp-deficient mice, dermal application of dexamethasone resulted in lower dermal and plasma concentrations, whereas epidermal concentrations were not reduced, supporting a spatially compartmentalized transporter contribution. Transdermal absorption of prednisolone was likewise reduced, whereas methylprednisolone and ethinyl estradiol were unaffected. In the same study, transporter-transfected cell assays identified dexamethasone and prednisolone as P-gp substrates, with only minimal BCRP-mediated transport, implicating P-gp as the principal transporter responsible for these compound-specific effects in this experimental system [85].

These findings define an important boundary for a purely diffusive interpretation of skin-permeation data. Synthetic membranes such as Strat-M^®^ and PDMS cannot recapitulate transporter-mediated processes because they lack viable cellular compartments and therefore capture only the passive, physicochemical components of transport. Conversely, transporter competence in viable reconstructed or bioprinted skin should be demonstrated functionally rather than inferred from cellular viability or transporter expression alone when such mechanisms are relevant to the compound under investigation. Transporter-mediated processes should therefore be regarded as compound- and compartment-specific modifiers of cutaneous disposition rather than as a general alternative to passive-diffusion models [82,84,85].

## 4. Cutaneous Distribution, Retention, and Local Exposure

In dermal pharmacokinetic studies, distribution becomes particularly relevant when the therapeutic or toxicological effect depends on local drug availability within the skin rather than on total receptor-phase permeation. Key distribution readouts include retention in the stratum corneum, accumulation in the viable epidermis, partitioning into the dermis, and, where relevant, localization within appendage-associated compartments. When downstream removal influences local tissue concentrations, transfer from the dermis into a receptor or vascular-like compartment provides an additional disposition readout, although clearance-like mechanisms are considered separately in Section 6. These endpoints are particularly important for topical dermatological products, for which local efficacy depends on drug delivery to the relevant epidermal or dermal target compartment and may not be adequately represented by receptor-phase permeation alone [2,21].

Retention values should be reported with an explicit normalization basis, as different normalizations address different questions and may alter model rankings. Amount per exposed area (µg cm^−2^) is most appropriate for formulation-delivery comparisons, whereas concentration per tissue mass or volume (e.g., µg g^−1^) better reflects tissue loading; percentage of applied dose supports mass balance interpretation, while percentage of recovered dose should be reported only together with the overall recovery. For epidermis-to-dermis partitioning, compartment concentrations are more informative than absolute amounts because the compartments differ in mass. As a minimum, studies should report the exposed area, applied dose, tissue-compartment mass and/or thickness, wet/dry or water-content basis, scaffold contribution for engineered constructs, and compartment-specific extraction recovery.

### 4.1. Synthetic Membranes

For distribution analysis, material-associated recovery from synthetic membranes must be distinguished from biological tissue retention. Strat-M^®^ and PDMS can be extracted after permeation experiments, but the recovered amount reflects partitioning or sorption within an artificial material rather than distribution within the stratum corneum, viable epidermis, or dermis. Membrane-associated drugs may nevertheless be relevant to analytical recovery and material-sorption assessment; for PDMS in particular, substantial partitioning of lipophilic compounds into the polymer can influence apparent exposure and transport kinetics [63]. Thus, membrane retention should not be interpreted as a surrogate for cutaneous tissue retention or layer-specific exposure.

### 4.2. Ex Vivo Human and Animal Skin

Ex vivo human and animal skin are the most informative conventional models for distribution analysis because they retain the layered architecture of native skin. Drugs can be quantified in the stratum corneum by sequential tape stripping, in the viable epidermis and dermis by tissue separation, sectioning, or spatial analytical methods, and in the receptor phase as an indicator of transdermal passage [2,13]. This compartmental resolution allows local tissue exposure to be distinguished from the drug that has traversed the skin and entered the receptor phase. However, the information obtained depends on tissue preparation: isolated epidermal membranes cannot resolve dermal retention, whereas dermatomed or full-thickness skin enables assessment of epidermal and dermal recovery [2,9,11,37]. The methodological strengths and limitations of the corresponding analytical approaches are considered in Section 4.7.

Full-thickness ex vivo skin is particularly informative when dermal retention is relevant because it allows differentiation among stratum-corneum retention, viable epidermal accumulation, dermal deposition, and receptor-phase passage [9]. However, the excised dermis lacks active blood and lymphatic flow and therefore acts as a static tissue reservoir rather than a physiologically cleared compartment. Receptor-phase recovery consequently represents transfer into an experimental sink rather than vascular clearance [2]. The implications of this limitation are considered in Section 6.

Animal skin introduces an additional translational limitation because compartment-specific retention may differ from human skin. Tissue thickness, follicular density, lipid distribution, and dermal composition vary among species, and drugs retained in porcine or rodent stratum corneum or dermis should therefore not be assumed to correspond quantitatively to human tissue exposure [4,71,72]. Porcine skin remains useful for comparative distribution studies, but species-specific differences should be considered when translating layer-specific retention to humans.

### 4.3. Reconstructed Human Epidermis

RHE models provide a simplified distribution space comprising a stratum corneum-like layer and viable epidermis. They can therefore support stratum-corneum sampling and measurement of epidermal drug uptake, but cannot assess dermal partitioning because they lack a dermal compartment [8,20]. Compounds traversing the epidermal construct enter the receptor compartment without encountering a true dermal reservoir, so receptor-phase appearance may occur without the delay or retention associated with partitioning into native dermal tissue. RHE is therefore most informative for questions involving stratum corneum or epidermal exposure, epidermal targeting, and comparative formulation assessment.

Because RHE generally differs from native human skin in both barrier function and tissue architecture, epidermal recovery should not be assumed to correspond quantitatively to native epidermal exposure [8,20,79]. Standard epidermis-only constructs are consequently less informative when dermal, follicular, or vascular exposure is the endpoint of interest.

### 4.4. Living Skin Equivalents and Full-Thickness Skin Equivalents

LSE/FTSE expands the accessible distribution space by adding a dermal equivalent beneath the epidermis. This enables assessment of epidermis–dermis partitioning and provides a tissue compartment in which dermal retention can be distinguished from epidermal uptake and receptor-phase permeation [65]. In the Phenion^®^ full-thickness model, Ackermann et al. demonstrated a dermal reservoir effect that was particularly evident for testosterone, showing that the dermal equivalent can materially influence tissue partitioning and receptor-phase appearance [65]. For questions centered on dermal retention, FTSE is therefore more informative than RHE because the dermal equivalent provides a pharmacokinetically relevant partitioning compartment rather than merely a structural spacer.

However, the dermal compartment in FTSE remains static. It can support tissue partitioning and retention, but does not reproduce perfusion-driven removal from the dermis. FTSE is therefore well suited to static epidermis–dermis distribution questions, whereas interpretation of vascular-like removal requires a perfused system; the underlying clearance-like mechanisms are discussed in Section 6.

### 4.5. Vascularized and Bioprinted Full-Thickness Constructs

Vascularized and bioprinted full-thickness constructs can extend distribution analysis beyond conventional epidermal and dermal compartments by incorporating vascular-like or appendage-like structures. Perfusable vascular compartments enable assessment of both topical-to-vascular and vascular-to-tissue distribution, whereas follicle-like structures provide an architectural basis for investigating appendage-associated localization and retention [7,81]. These capabilities may therefore be particularly relevant when the distribution endpoint involves vascular-side exposure, dynamic dermal removal, or follicular targeting.

However, architectural representation should not be equated with validated pharmacokinetic function. Quantitative benchmarks for layer-specific retention, follicular uptake, and vascular recovery remain sparse compared with EHS, porcine skin, and conventional Franz-cell IVPT, and transfollicular transport in follicle-containing engineered constructs remains insufficiently validated [2,7,81]. These systems are therefore currently best regarded as mechanistic platforms for compartment-resolved distribution rather than standardized models for quantitative tissue-partitioning prediction.

### 4.6. Perfused and Microfluidic Skin-on-Chip Systems

From a distribution perspective, the main value of perfused and microfluidic systems is their ability to couple tissue retention with dynamic downstream transfer. By continuously removing the drug from the basal side, these platforms can shift the balance between dermal accumulation and transfer into a vascular-like output, thereby allowing tissue exposure to be interpreted together with time-resolved downstream recovery [6,7,33,34,80]. This distinction is particularly relevant when local tissue concentrations are influenced by ongoing removal from the dermal compartment. The observed distribution profile nevertheless remains dependent on the architecture and barrier properties of the integrated skin model. Detailed receptor-side transport and clearance-like mechanisms are discussed in Section 6.

### 4.7. Analytical Methods for Depth- and Compartment-Resolved Distribution

Depth- and compartment-resolved measurements complement receptor-phase permeation by identifying where a topically applied compound resides within the skin. To place in vitro distribution data in a translational context, selected in vivo methods are also considered here as reference approaches for local cutaneous exposure.

Tape stripping is an established method for assessing drug disposition in the stratum corneum (SC). Quantitative interpretation requires accounting for the amount of SC removed rather than relying on the tape-strip number alone. In the studies by Herkenne et al. [86,87], the SC collected on the tape strips was quantified gravimetrically, while transepidermal water loss measurements were used to estimate the apparent total SC thickness and to express drug concentration as a function of relative SC depth. The resulting concentration–depth profiles were fitted mathematically to derive an SC–vehicle partition coefficient, a first-order kinetic parameter related to drug diffusivity, and the total amount of drug present in the SC at the end of application [86,87]. In a later two-investigator study using a standardized tape-stripping protocol, drug-mass measurements were generally reproducible, although residual measurement variability remained and could necessitate larger study populations [22].

For ibuprofen in defined propylene glycol–water vehicles, Herkenne et al. [86] found that partitioning- and diffusion-related parameters obtained using ex vivo pig-ear SC were broadly comparable with those measured in vivo in humans following a 30 min application. Parameters derived from the 30 min experiments were subsequently used to predict SC exposure after three hours, with no significant differences between the predicted and experimentally measured AUC values [86]. In a subsequent comparison of four marketed ibuprofen formulations, in vitro permeation across freshly excised pig skin correlated better with human in vivo dermatopharmacokinetic results than permeation across a silicone membrane [87]. These findings support quantitative tape stripping and ex vivo pig-ear skin for formulation comparisons under defined experimental conditions, but do not establish universal equivalence between tape stripping and IVPT. Tape stripping quantifies the drug associated with successive SC layers, whereas the conventional IVPT receptor-phase endpoint quantifies the drug that has crossed the skin into the receptor compartment.

Confocal Raman spectroscopy (CRS) provides a complementary, label-free, and non-invasive approach to chemical depth profiling. In human volunteers, Mateus et al. showed that CRS-derived ibuprofen distribution profiles within the SC were comparable with corresponding tape-stripping profiles [88]. Ex vivo studies have further demonstrated that CRS can interrogate drug disposition below the SC, including time-resolved uptake and depletion or redistribution within the viable epidermis; however, Raman signal attenuation increases with tissue depth and requires appropriate correction and normalization for quantitative interpretation [89].

Mass spectrometry imaging (MSI) adds molecularly specific spatial information across skin sections. Matrix-assisted laser desorption/ionization (MALDI)-MSI has been used to generate quantitative distribution profiles of topically applied drugs in human skin explants and to distinguish formulation-dependent penetration patterns [90,91]. Such quantification requires appropriate calibration within the tissue context rather than interpretation of raw signal intensity alone. Bonnel et al., for example, based quantitative profiles on tissue-extinction-coefficient calibration [90]. MSI can also provide spatially resolved functional information: Couto et al. used substrate-based MSI to demonstrate esterase activity in the LabSkin living skin equivalent, alongside proteomic comparison with ex vivo human skin [24].

A non-imaging, destructive complement is the cutaneous biodistribution method (CBM), in which a frozen skin disc is serially cryosectioned parallel to the skin surface, and the analyte recovered from each lamella is quantified to generate an amount-versus-depth profile extending from the stratum corneum through the viable epidermis into the upper dermis. Gou et al. analyzed twenty 20 µm lamellae per disc, corresponding to a depth of approximately 400 µm, and obtained comparable biodistribution profiles when cryosectioning was performed by three skilled operators at two laboratories. Because the IVPT exposure, sample extraction, and ultra-high-performance liquid chromatography–tandem mass spectrometry (UHPLC–MS/MS) quantification were centralized, the study supports the intra- and inter-laboratory reproducibility of the cryosectioning component under the standardized conditions tested, rather than complete end-to-end interlaboratory reproducibility of the CBM. CBM should also be distinguished from imaging approaches: because each lamella is extracted and analyzed in bulk, it resolves distribution with depth but does not retain lateral or cellular localization. Selection among imaging modalities should be guided by the biological question and the required spatial and temporal resolution, as well as sensitivity, chemical specificity, imaging depth, analyte compatibility (including the need for labeling), and whether ex vivo or in vivo measurements are required [92,93].

Dermal microdialysis provides a different pharmacokinetic readout by serially sampling the accessible extracellular drug fraction within the dermal interstitial space. In healthy volunteers, dermal microdialysis generated time-resolved lidocaine exposure profiles and discriminated between topical cream and ointment formulations, with approximately three- to five-fold higher dermal dialysate exposure from the cream [94]. However, dialysate concentration represents only a fraction of the extracellular concentration; quantitative interpretation therefore requires determination of probe recovery, for example, by in situ calibration or retrodialysis [95]. Dermal open-flow microperfusion (dOFM) provides a membrane-free alternative to dermal microdialysis. The dOFM probe contains macroscopic openings and is operated in push–pull mode, thereby circumventing the molecular-weight cut-off and other recovery limitations imposed by the semipermeable membrane. This design enables continuous sampling of diluted, unfiltered dermal interstitial fluid and facilitates assessment of analytes that may be poorly recovered by membrane-based probes, including lipophilic and higher-molecular-weight substances [96]. Nevertheless, membrane-free sampling should not be interpreted as a complete recovery of undiluted interstitial fluid; exchange stability or relative recovery may still require evaluation, particularly when absolute tissue concentrations are sought [97]. In a direct comparison using ex vivo human skin, dOFM and dermal microdialysis yielded concordant pharmacokinetic results for topically applied fentanyl and benzoic acid under the conditions investigated, although both methods retained technique-specific advantages and limitations [98].

Compartment-resolved retention measurements are also susceptible to sample-preparation artifacts and cross-contamination. Incomplete removal of residual surface formulation may inflate apparent stratum corneum or epidermal retention, whereas sectioning, heat separation, follicular residues, and freezing or embedding may redistribute compound between nominal tissue compartments. Studies should therefore report and, where appropriate, validate the washing procedure; determine extraction recovery separately for each analyzed layer; verify successful tissue separation by histology or suitable layer-specific markers; and include processing blanks and carryover controls. Where these controls are incomplete, compartment-specific retention should be interpreted cautiously because apparent epidermal or dermal drug levels may partly reflect preparation-related contamination rather than true tissue distribution.

Accordingly, these methods are complementary rather than interchangeable because they interrogate distinct physical and pharmacokinetic compartments: tape stripping measures SC-associated drug; CRS provides chemical depth profiles; MSI maps spatial molecular distribution across tissue sections; CBM provides destructive, depth-resolved quantification across serial skin lamellae, and microdialysis and dOFM provide time-resolved extracellular dermal exposure using membrane-based and membrane-free sampling, respectively.

## 5. Metabolism: Local Biotransformation and Model-Specific Metabolic Competence

Metabolic competence is an important discriminator among in vitro skin models because it determines whether a system can capture local biotransformation, metabolite formation, detoxification, or metabolic activation of topically applied compounds. This is particularly relevant when efficacy or toxicity depends on cutaneous metabolism. Model selection should therefore consider not only barrier properties but also whether the system contains viable cells with demonstrable pathway-relevant activity and compound-specific metabolite formation.

In studies of viable skin models, metabolic competence should be interpreted alongside tissue viability. In particular, the absence or low metabolite formation cannot be confidently attributed to limited pathway activity unless tissue viability is maintained throughout the relevant exposure period. Terminal viability assessment should therefore accompany compound-specific functional readouts and, where appropriate, pathway-relevant positive controls. Importantly, viability alone does not demonstrate metabolic competence; functional evidence of biotransformation remains necessary. Non-detection of a metabolite is also conditional on analytical performance and should not by itself be interpreted as evidence of absent metabolism. With LC–MS, inadequate chromatographic separation, inappropriate ionization conditions, low ionization efficiency, or matrix effects may result in false-negative findings. Studies interpreting the absence of metabolism should therefore report the analytical method and its sensitivity for the expected metabolites, including relevant detection or quantification limits where these can be established, and should use pathway-relevant positive controls to demonstrate metabolite detectability where appropriate [60].

Parent-compound depletion is likewise not specific evidence of metabolism, because parent-compound loss may reflect chemical instability, abiotic hydrolysis, adsorption, incomplete extraction, or nonspecific uptake or binding. Claims based on depletion should, where applicable, be supported by cell- or tissue-free stability controls under matched conditions, time-dependent metabolite formation above the abiotic background, parent-plus-metabolite mass balance where analytically feasible, and a pathway-relevant positive control. In the absence of such controls, parent depletion should be interpreted as consistent with, rather than demonstrative of, cutaneous metabolism.

### 5.1. Synthetic Membranes

With respect to metabolism, synthetic membranes have no intrinsic metabolic capacity. Consequently, although they remain useful for physicochemical permeation testing, they cannot reproduce parent-compound biotransformation, metabolite formation, detoxification, or metabolic activation within the skin [2,25]. For metabolism-sensitive compounds, these systems can therefore provide information on passive transport or release but not on local cutaneous biotransformation.

### 5.2. Ex Vivo Human Skin

For metabolism studies, ex vivo human skin represents the closest native reference among isolated skin models because it retains the endogenous cutaneous xenobiotic-metabolizing enzyme repertoire. Fresh full-thickness skin explants have demonstrated functionally relevant phase II pathways, including glucuronidation, sulfation, N-acetylation, catechol-O-methylation, and glutathione conjugation [26,99]. However, metabolic competence after excision depends strongly on tissue handling and storage, and loss of viability does not abolish all enzymatic activity. Esterase-mediated hydrolysis can persist in previously frozen human skin [100], whereas oxidative and conjugative pathways are generally more dependent on viable tissue [26,99]. Frozen or otherwise non-viable skin should therefore not be considered metabolically inert, and hydrolytic conversion should be assessed directly for susceptible compounds, including esters and peptides [100,101]. Fresh tissue remains preferable when metabolism is a primary endpoint, and terminal viability should be documented when metabolite formation, or its absence, is used to infer metabolic competence.

Donor source, anatomical site, and tissue handling introduce additional variability, so ex vivo human skin should be regarded as the closest native reference rather than a fully standardized metabolic system.

### 5.3. Ex Vivo Animal Skin

In contrast to their use as permeability surrogates discussed in Section 3.3, the metabolic relevance of ex vivo animal skin is determined by species- and pathway-specific xenobiotic-metabolizing activity. Porcine skin can serve as a useful comparative model, and direct human–pig explant studies have demonstrated overlapping metabolism among several test compounds. Nevertheless, animal skin should not be considered a quantitatively predictive substitute for human cutaneous metabolism without compound- and pathway-specific validation [25,27].

### 5.4. Reconstructed Human Epidermis and Full-Thickness Skin Equivalents

Beyond their established use in permeation studies (Section 3.4), RHE and FTSE can support investigation of selected pathways of cutaneous metabolism. Their metabolic competence, however, is pathway-dependent and should not be assumed to be globally equivalent to that of native skin or liver. Across available studies, a consistent pattern is low basal cytochrome P450 (CYP)-dependent activity together with better preservation of several non-CYP phase I and phase II pathways. Götz et al. found no detectable EROD, MROD, PROD, or 7-methoxy-4-trifluoromethylcoumarin O-dealkylase activity in EpiDerm^®^ microsomes and only very low CYP3A activity. However, intact EpiDerm^®^ exhibited inducible CYP1-associated activity following 3-methylcholanthrene exposure, indicating that selected inducible responses may persist despite minimal basal CYP activity [102]. Consistent with this profile, Jäckh et al. detected no measurable CYP activity in EpiDerm^®^ or Phenion^®^ FT despite evidence of CYP gene expression, while confirming flavin-containing monooxygenase (FMO), N-acetyltransferase (NAT), and UDP-glucuronosyltransferase expression and activity in both models [28].

Luu-The et al. similarly reported broad phase I/II enzyme expression in EpiSkin^®^ and its full-thickness counterpart, with low CYP and FMO expression but stronger representation of phase II enzymes, including GSTP1, COMT, SULT2B1b, and NAT5 [103]. Because these findings were based primarily on mRNA expression, they support the biological plausibility of the corresponding pathways but do not, by themselves, establish functional metabolic competence. This distinction is critical: enzyme expression should be complemented by functional evidence such as substrate depletion, metabolite formation, or pathway-specific activity.

Proteomic and functional studies provide further evidence for preserved non-CYP metabolism in reconstructed skin. Couto et al. found a similar abundance of ADH1C and GSTP1 in the LabSkin living skin equivalent and ex vivo human skin, quantified several esterases, and confirmed esterase activity using substrate-based mass spectrometry imaging. In contrast, CYP activity was not detected for the tested substrates, consistent with the absence of the corresponding CYP proteins [24]. Wiegand et al. further demonstrated this organ-specific metabolic profile, with phase I metabolism being more pronounced in a liver model and phase II metabolism more prominent in reconstructed skin models [29].

Overall, RHE and FTSE are useful for selected cutaneous metabolic pathways, particularly those associated with flavin-containing monooxygenase, N-acetyltransferase, UDP-glucuronosyltransferase, glutathione S-transferase, alcohol dehydrogenase, and esterase enzymes. Their metabolic competence should nevertheless be established for the specific pathway and compound under investigation, and broad liver-like CYP-dependent activation should not be assumed without direct functional evidence.

### 5.5. Bioprinted Full-Thickness Constructs

Metabolic competence in bioprinted full-thickness skin remains insufficiently characterized. Although bioprinting can provide more complex cellular and tissue architectures and may be combined with vascular-like compartments or perfusion, these features do not in themselves establish functional xenobiotic metabolism. Metabolic competence should therefore be demonstrated for the specific construct and compound using pathway-relevant functional endpoints, such as parent-compound depletion and metabolite formation, rather than inferred solely from architectural complexity or cell viability.

### 5.6. Skin-on-Chip and Coupled Organ Systems

For skin-on-chip systems, an important distinction should be made between local metabolism within the skin compartment and metabolism arising from coupled organs. Skin-only platforms may reproduce cutaneous biotransformation when they incorporate metabolically competent reconstructed or ex vivo skin, although compound-specific metabolite formation has rarely been the primary pharmacokinetic endpoint [25,26]. Coupled skin–liver systems extend this approach by enabling simultaneous investigation of skin permeation, cutaneous first-pass metabolism, and downstream systemic metabolism [104,105].

HUMIMIC Skin–Liver Chip2 studies combined reconstructed skin models with liver spheroids to investigate 4-amino-2-hydroxytoluene following topical or systemic application. Tao et al. demonstrated application-route-dependent metabolism of 4-amino-2-hydroxytoluene and recapitulated aspects of the cutaneous first-pass effect observed in vivo. Brandmair et al. further demonstrated that the choice of reconstructed skin model affected both permeation and metabolism: epidermal models metabolized 4-amino-2-hydroxytoluene, whereas PhenionFT^®^ showed slower permeation, closer to native human skin, together with extensive formation of N-acetyl-4-amino-2-hydroxytoluene [104,105]. These findings illustrate the particular value of coupled systems when local skin metabolism and downstream systemic metabolism jointly determine the pharmacokinetic profile.

Overall, metabolic relevance varies fundamentally among in vitro skin models. Synthetic membranes have no intrinsic metabolic capacity, whereas ex vivo human skin remains the closest native reference. Animal skin may support comparative metabolic studies but requires compound- and pathway-specific assessment of human relevance. RHE and FTSE provide reproducible human-cell-based platforms with useful non-CYP, phase II, and esterase-related activity but generally weak basal CYP activity, whereas metabolic competence in bioprinted and skin-on-chip systems remains more model-specific. Across all viable platforms, metabolic capability should therefore be demonstrated functionally rather than inferred from tissue complexity, cellular viability, or enzyme expression alone.

## 6. Cutaneous Clearance and Clearance-like Removal

### 6.1. Static Diffusion-Cell Systems

Clearance-like removal represents one of the clearest pharmacokinetic distinctions between static and dynamic in vitro skin models. Static systems, including synthetic membranes, ex vivo skin, RHE, and conventional FTSE mounted in Franz diffusion cells, can quantify permeation into a receptor phase and support retrospective mass balance analysis across donor, tissue, and receptor compartments, as described in OECD TG 428 and its guidance notes [9]. However, even when receptor-side sink conditions are maintained, static receptor sampling does not reproduce active dermal vascular or lymphatic clearance. Transfer from viable cutaneous tissue into the receptor phase should therefore be regarded as an experimental approximation of downstream removal rather than physiological clearance [31].

In Franz-cell experiments, receptor-phase permeation depends strongly on maintenance of sink conditions, which is influenced by receptor medium composition, receptor volume, sampling strategy, and drug solubility. Loss of sink conditions is particularly problematic for poorly soluble or lipophilic compounds, for which receptor accumulation or insufficient solubilization may distort apparent flux and permeability. Baert et al. demonstrated this for testosterone, showing that receptor medium composition affected assay discrimination and that hydroxypropyl-β-cyclodextrin-containing media performed best because of their greater solubilization capacity [2,32]. Optimizing receptor conditions can therefore minimize the methodological limitations of static diffusion cells, but should not be interpreted as a means of reproducing physiological clearance.

### 6.2. Perfused and Microfluidic Systems

Perfused and microfluidic skin models address part of this limitation by continuously renewing the receptor-side medium, thereby maintaining lower downstream concentrations and more closely approximating clearance-like removal than a static receptor chamber. In the vascularized construct described in Section 3.5, a perfused vascular network enabled both topical-to-vascular and vascular-to-tissue transport, demonstrating the pharmacokinetic potential of a dynamic vascular-like compartment [7]. Prolonged perfused culture has also been demonstrated in a bioprinted vascularized construct, establishing technical feasibility, although validation against quantitative clearance-related endpoints remains necessary [80].

Microfluidic permeation platforms provide a particularly direct methodological basis for dynamic receptor renewal (Section 3.6) [33]. Importantly, flow-through conditions also change how transport is quantified and require that the quantity reported be named precisely. Rather than deriving flux from cumulative accumulation within a fixed receptor volume, transport is calculated from the perfusate concentration and the volumetric flow rate. The primary measurement is the receptor-side appearance rate, that is, the amount of compound leaving the system per unit time [106].

(7) $$\frac{dM}{dt}=Q_{f}\left(C_{out}-C_{in}\right)$$

where dM/dt is the appearance rate (µg h^−1^), Qf is the volumetric flow rate (µL h^−1^ or mL h^−1^), and Cout and Cin are the compound concentrations in the outlet and inlet perfusate. Cin is zero only where fresh medium is delivered continuously; in a recirculating configuration, the inlet concentration rises during the experiment and must be measured at each sampling point rather than assumed to be negligible.

Because the appearance rate depends on the exposed area of a particular device, it is not comparable across platforms with different geometries. Normalization to the exposed area gives the area-normalized flux, which is the quantity directly comparable with flux measured in a static diffusion cell:

(8) $$J\;=\;\frac{Q_{f}\left(C_{out}\;-\;C_{in}\right)}{A}$$

where A is the exposed diffusion area (cm^2^), and J has units of µg cm^−2^ h^−1^.

Neither of these quantities is a clearance. Where a clearance-like parameter is required, it should be reported as a concentration-normalized quantity, that is, normalized to the concentration in the compartment from which the compound is removed:

(9) $${CL}_{app}\;=\;\frac{Q_{f}\left(C_{out}\;-\;C_{in}\right)}{C_{ref}}$$

where Cref is the concentration in that source compartment. CLapp has units of volume per unit time (µL h^−1^) and is the only one of these three expressions with the dimensions of pharmacokinetic clearance. The identity of Cref must be stated, since values normalized to the donor concentration, to the receptor-side tissue concentration, or to the concentration in a vascular-like compartment answer different questions and are not interchangeable. Receptor-fluid replacement, continuous flow, basal transport, and perfusate recovery are experimental means of achieving removal; reporting them as clearance without this normalization implies a physiological equivalence that the measurement does not support.

**Figure 3:**
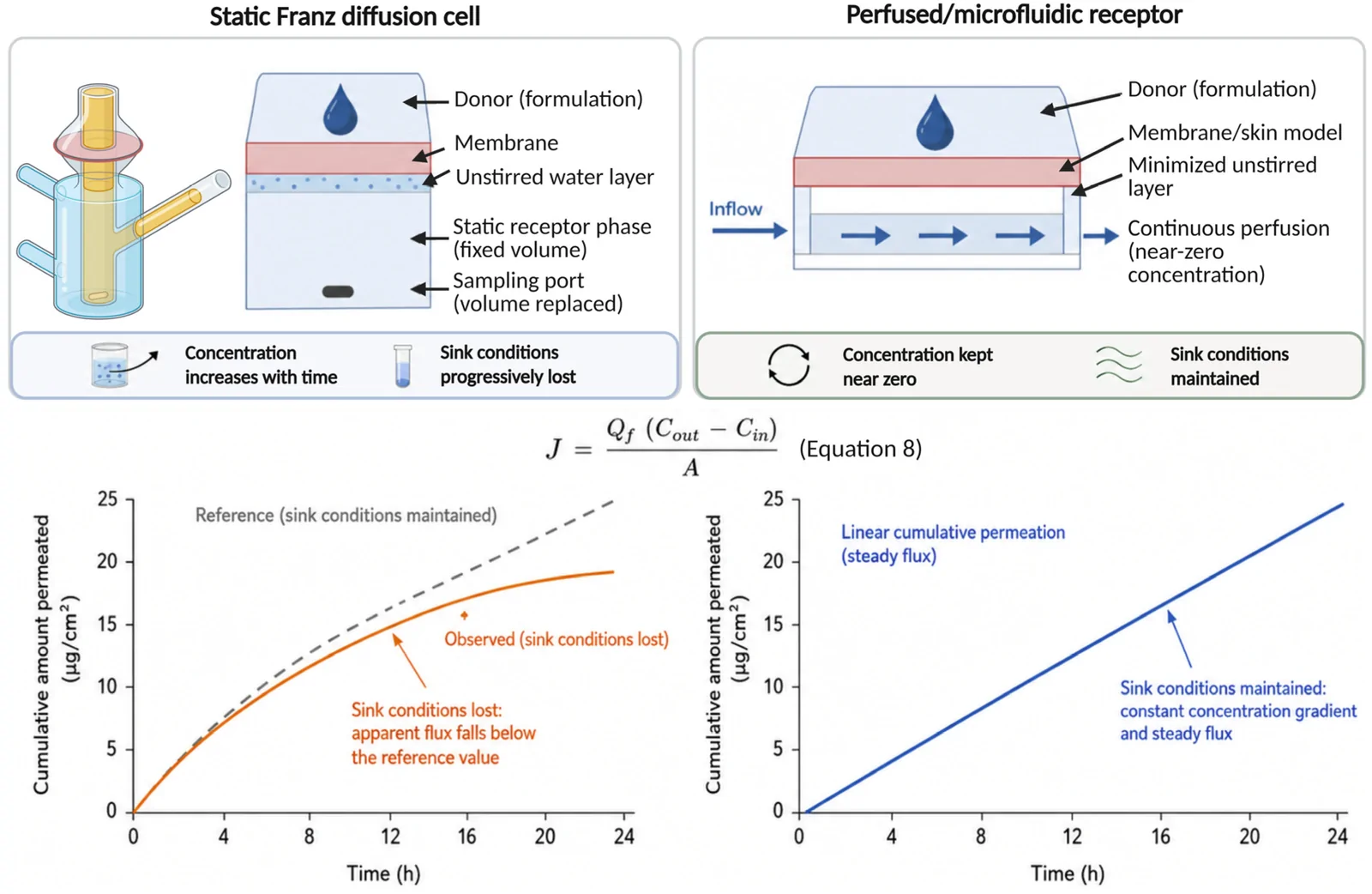
Static and dynamic receptor conditions. In a static Franz diffusion cell, the receptor volume is fixed, an unstirred water layer may develop beneath the barrier, and receptor concentration increases over time; in the illustrative sink-limited scenario shown, apparent flux decreases relative to otherwise comparable sink-controlled conditions (dashed line). In a perfused or microfluidic receptor, continuous medium renewal maintains a low downstream concentration, reduces receptor-side accumulation and boundary-layer effects, and maintains the concentration gradient. Under these conditions, the receptor-side appearance rate is obtained from the volumetric flow rate, and the difference between the outlet and inlet perfusate concentrations, and the area-normalized flux J = Qf(Cout−Cin)/A follows by division by the exposed area A (Section 6.2, Equations (7) and (8)); Cin is zero only where fresh medium is delivered continuously. Curves are illustrative. “Static and dynamic receptor conditions”created in BioRender. Maver, U. (https://BioRender.com/o91axpi) is licensed under CC BY 4.0.

Continuous renewal can maintain low downstream concentrations and reduce the risk that receptor-side accumulation becomes rate-limiting (Figure 3). These advantages are not automatic, and several device-level factors determine whether the calculated values reflect transport across the barrier or the behavior of the fluidic system itself. The configuration must be stated: in a single-pass system, the perfusate passes the tissue once, and the inlet concentration is that of fresh medium, whereas in a recirculating system, the inlet concentration rises during the experiment, sink conditions deteriorate progressively, and the driving force is not constant. System dead volume and the residence time between the tissue interface and the collection point introduce a transit delay between transport and detection, so that an apparent tlag measured in a perfused device contains a fluidic component; this should be measured, for example, with a non-retained tracer, and subtracted before the remainder is interpreted as a barrier property. The sampling interval should be matched to that residence time, since sampling more frequently than the system can resolve produces apparent structure that is an artifact of dispersion within the channels. Limited solubility of the compound in the perfusate, or an inadequate flow rate, can reintroduce the downstream transport limitation that continuous renewal is intended to remove. Where the perfusate contains protein, only the unbound fraction is available for transport and for partitioning back into the tissue, so protein binding should be characterized and reported alongside the medium composition. In vascularized constructs, the endothelial layer contributes its own resistance in series with the skin, so the measured output reflects barrier and endothelium together and cannot be attributed to the skin alone without a separate endothelium-only control. Finally, adsorption onto channels and tubing, particularly for lipophilic compounds in devices containing bulk PDMS, removes the compound from the measured stream, thereby depressing apparent flux and breaking mass balance; a compound-specific device recovery experiment performed without tissue is therefore a necessary control for any quantitative perfusion measurement. Perfused systems are therefore particularly informative when receptor-side resistance, tissue retention, or compound lipophilicity materially affects apparent permeability in static assays.

Overall, no in vitro skin model fully reproduces physiological dermal clearance. Static diffusion-cell systems provide useful receptor-phase permeation and mass balance data but lack dynamic vascular or lymphatic removal. Perfused and microfluidic platforms provide a closer experimental approximation by coupling tissue release to continuous downstream removal and time-resolved sampling. Their principal pharmacokinetic value lies not in reproducing physiological clearance itself, but in reducing receptor-side artifacts and providing a dynamic framework for interpreting flux, tissue release, and vascular-side exposure.

### 6.3. Desquamation and In Vivo Benchmarks for Cutaneous Clearance

A physiologically relevant removal mechanism that is largely absent from routine IVPT and short-duration in vitro skin experiments is the continuous renewal and desquamation of the stratum corneum. In vivo, terminally differentiated corneocytes are progressively shed from the skin surface and replaced by newly formed cells, providing an outward route by which compounds retained within the superficial stratum corneum may ultimately be removed. Desquamation, therefore, competes with inward diffusion: a compound residing within the stratum corneum may either continue to diffuse toward the viable epidermis or remain sufficiently superficial to be lost with shed corneocytes. Mathematical analyses of percutaneous transport have shown that epidermal turnover can influence both drug concentrations within the skin and the kinetics of systemic absorption, while experimental work has described desquamation as a counter-flux that becomes increasingly relevant when inward permeation is slow relative to stratum corneum renewal [107,108].

This physiological process should be distinguished from the “clearance from the stratum corneum” measured in dermatopharmacokinetic tape-stripping studies. In these experiments, the formulation is removed after a defined uptake period, and the subsequent decrease in stratum corneum drug content is measured at defined clearance times. Nicoli et al. demonstrated that this post-application clearance phase is governed primarily by drug diffusion within the stratum corneum, whereas Hoppel et al. showed that the resulting depletion kinetics can be used to estimate the rate at which the drug is delivered from the stratum corneum into the underlying viable skin [21,109]. Thus, over the timescale of these experiments, a decrease in stratum corneum drug content should be interpreted primarily as a loss due to inward diffusion rather than to physiological desquamation.

This distinction is important when interpreting tissue-retention measurements from conventional in vitro studies. Routine diffusion-cell experiments, typically conducted over hours to one or two days, do not reproduce the longer-timescale physiological renewal of the stratum corneum. Consequently, the drug recovered from the stratum corneum at the end of an experiment represents an experimentally retained fraction rather than a direct measure of how long that reservoir would persist in vivo. This limitation is particularly relevant when inward diffusion is slow, and a substantial fraction of the applied compound remains within superficial stratum corneum layers. Regulatory dermal-absorption guidance recognizes this uncertainty: material retained in the upper stratum corneum may be lost by desquamation and, under defined conditions, excluded from the absorbable fraction, whereas material in deeper stratum corneum layers is generally considered potentially available for subsequent absorption unless evidence indicates otherwise [11]. Consistent with this approach, Pieper et al. reported that under conditions of incomplete absorption, drug recovered from tape strips beyond the first two is included in the absorbed fraction, whereas the first two strips are excluded; when absorption is sufficiently complete over a 24 h sampling period, all tape-strip fractions may be excluded [110].

Desquamation is not the only route by which compounds leave the stratum corneum in vivo. A soap-based washing protocol has been shown to extract caffeine as deep as the tenth tape strip, and sweating, bathing, and rubbing against clothing act in the same direction [111]. None of these is reproduced in a covered diffusion cell, so in vitro stratum corneum retention represents an upper bound relative to a realistic exposure scenario, and the washing protocol should be reported as part of that scenario rather than as a sample-preparation step.

A complementary in vivo benchmark is provided by cutaneous microdialysis, which reflects drug disposition within the viable dermis rather than the stratum corneum. Morgan et al. investigated the hydrophilic antivirals aciclovir and penciclovir and demonstrated that dermal drug concentrations were determined not only by stratum corneum resistance but also by local microvascular perfusion. Reducing cutaneous blood flow markedly increased dermal drug recovery, demonstrating that the microcirculation can remove absorbed drug and thereby limit local tissue concentrations [112]. This coupling between barrier penetration and vascular removal is absent from conventional static diffusion cells, in which receptor-phase accumulation represents transfer into an experimental sink rather than physiological microvascular clearance.

Desquamation and vascular removal, therefore, represent two spatially distinct determinants of cutaneous drug disposition: desquamation can remove drug retained within the superficial stratum corneum toward the skin surface, whereas dermal microvascular perfusion removes drug after it reaches viable tissue. Conventional static IVPT reproduces neither process physiologically. Perfused and microfluidic systems provide a closer approximation of the vascular-side component but generally do not reproduce physiological stratum corneum turnover. Accordingly, endpoint tissue retention and receptor-phase accumulation should not be interpreted as direct surrogates for in vivo persistence or dermal concentration without considering these compartment-specific removal mechanisms.

## 7. From In Vitro Measurement to Regulatory Use: Mechanistic Extrapolation and Current Acceptance

### 7.1. Mechanistic In Vitro–In Vivo Extrapolation and Dermal PBPK Modeling

**Figure 4:**
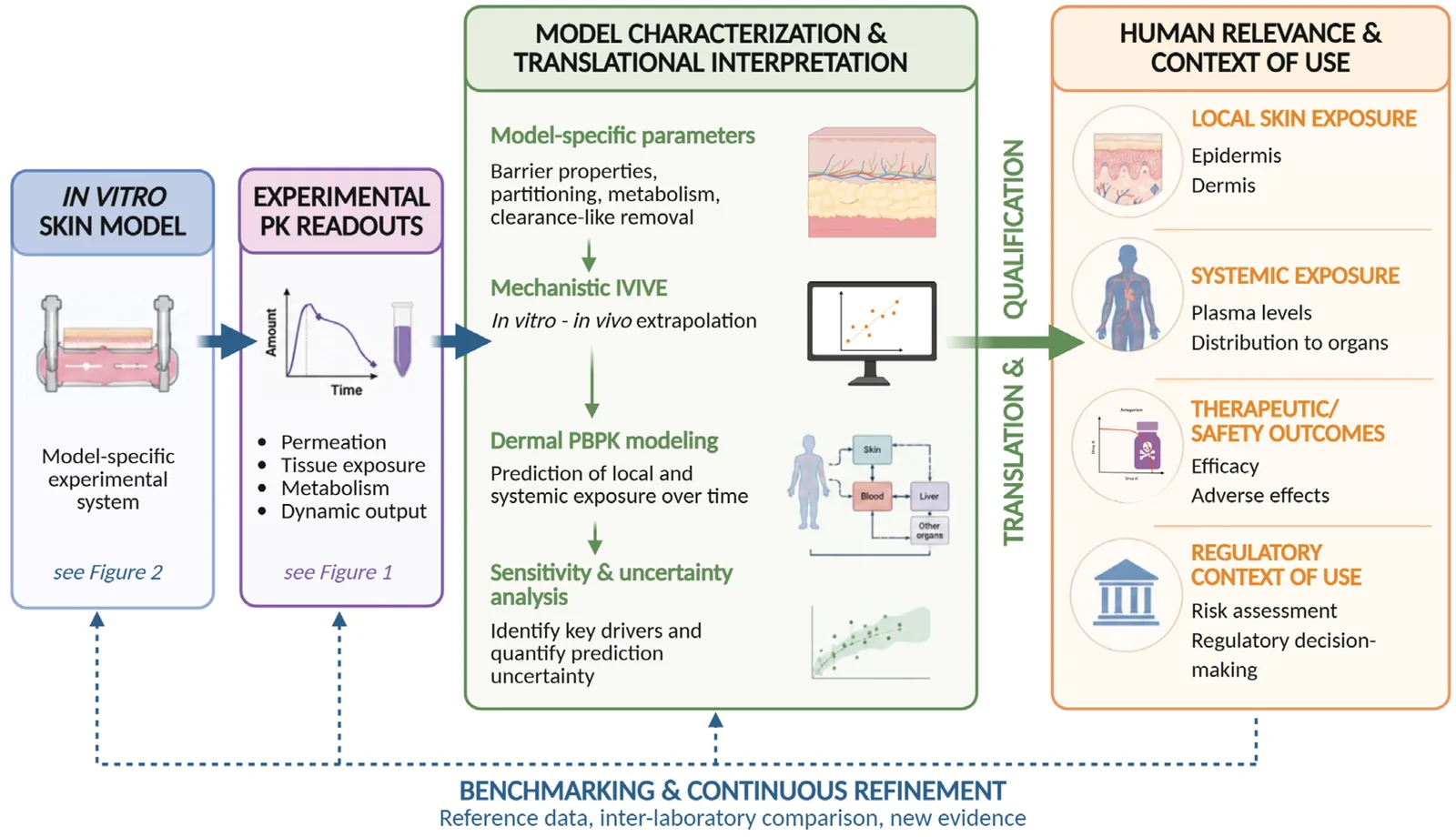
Conceptual framework for translating in vitro skin-model measurements toward human-relevant pharmacokinetic interpretation. Model-specific experimental outputs (Figure 1 and Figure 2) serve as inputs for mechanistic IVIVE and dermal PBPK modeling, supported by model characterization and sensitivity and uncertainty analyses. These approaches can inform estimates of local and systemic exposure and their interpretation in therapeutic, safety, and regulatory contexts. Benchmarking against reference data and inter-laboratory evidence provides a basis for continuous model refinement. The framework does not imply that all model classes support every endpoint or are qualified for regulatory use. “Conceptual framework for translating in vitro skin-model measurements toward human-relevant pharmacokinetic interpretation”created in BioRender. Maver, U. (https://BioRender.com/bhgc43q) is licensed under CC BY 4.0.

In vitro permeation values are inherently model-specific and therefore require a translational step before they can be interpreted in relation to human exposure (Figure 4).

Mechanistic dermal PBPK modeling provides one framework for this translation. Layered diffusion–partition models represent the stratum corneum, viable epidermis, and dermis as sequential compartments and allow experimentally derived IVPT data to inform parameters that would otherwise be estimated in silico [66,67].

The stratum-corneum: vehicle partition coefficient has emerged as an influential sensitivity parameter in several mechanistic dermal models, and optimization against experimental data has improved predictions of human systemic exposure in reported applications, including models informed by porcine skin data [68]. An additional difficulty applies under finite dosing, where the vehicle itself changes through evaporation, water uptake, and compositional shifts, so that this partition coefficient is time-varying rather than constant [113]. Models parameterized with a single static value may therefore misrepresent finite-dose exposure even where they reproduce infinite-dose data well, and formulation metamorphosis should be treated as an explicit source of parameter uncertainty in dermal PBPK applications. IVPT-informed models can also extend prediction beyond systemic exposure to local tissue concentrations. For metronidazole, for example, an IVPT-informed model reproduced clinically measured stratum corneum amounts [70]. Conversely, bottom-up mechanistic models can help interpret variability observed within IVPT datasets and identify the processes most likely to contribute to experimental differences [69].

The translational implication is that systematic and well-characterized differences between an experimental barrier and human skin may, in selected contexts, be addressed through model-based calibration. This possibility should not, however, be interpreted as implying that any model-specific bias is readily correctable. Deviations that vary with compound, formulation, or experimental conditions, particularly when they change in magnitude or direction, are substantially more difficult to translate and are unlikely to be captured by a single universal scaling factor; any correction should therefore be defined and validated within a stated applicability domain.

### 7.2. Regulatory Qualification and Current Context of Use

Regulatory acceptance of in vitro skin models is endpoint- and context-specific, and three distinct applications should be differentiated: hazard identification, dermal absorption assessment, and topical bioequivalence. For hazard identification, RHE models are validated under OECD TG 431 and TG 439 for skin corrosion and irritation, respectively [114,115]. These methods rely primarily on viability-based endpoints, and their regulatory validation should not be interpreted as qualification for quantitative drug transport or pharmacokinetic assessment.

For dermal absorption, OECD TG 428 and Guidance Document 156 are centered on excised human or animal skin and recognize that tissue preparation can materially affect measured absorption, including potential overestimation when epidermal membranes are used [9,11]. The use of reconstructed models remains conditional on demonstrating appropriate comparability with reference chemicals and does not constitute a general regulatory qualification as a replacement for native skin [11]. This distinction is particularly important given the model- and compound-dependent permeability differences discussed in Section 3.4.

For topical bioequivalence, current guidance remains centered on ex vivo human skin. The FDA draft IVPT guidance specifies ex vivo human skin and recommends 4–6 pilot donors with at least four replicate skin sections per donor per treatment group [10]. The EMA guideline on quality and equivalence of locally applied, locally acting cutaneous products, adopted by the CHMP in September 2024 and applicable since April 2025, requires that the dosing amount be based on the posology of the reference product, that dose application be validated for reproducibility within ±5%, and that the donor compartment be unoccluded unless the product information specifies otherwise [12]. The same guideline includes in vivo tape stripping among the permeation kinetic methods accepted in lieu of a clinical equivalence study, alongside in vitro skin permeation and pharmacokinetic bioequivalence, but its use is conditional: it is reserved for compounds that do not permeate through the skin yet show quantifiable diffusion across the stratum corneum, whereas in vitro skin permeation is preferred where the compound reaches the receptor phase in quantifiable amounts [12]. Dermal microdialysis, open-flow microperfusion, and confocal Raman spectroscopy are named in the same guideline as not currently sufficiently established to provide pivotal evidence, although they may be used supportively and could become pivotal if appropriately qualified [12]. The corresponding FDA guidance on IVPT studies for topical products submitted in abbreviated new drug applications remains in draft form [10], whereas FDA guidance on physicochemical and structural (Q3) characterization of topical drug products was finalized in March 2026 [44]. Reconstructed, bioprinted, and perfused skin platforms are not currently considered pivotal barriers in these quantitative IVPT applications [10,12].

Importantly, however, comparative product assessment poses a different question from absolute prediction of human permeability. A model that differs systematically from native human skin may still provide useful discriminatory information if it reproducibly distinguishes formulation-dependent performance under matched conditions. Modified IVPT using RHE, for example, discriminated among clotrimazole creams containing 50%, 100%, and 200% of nominal drug strength without placebo interference [79]. Such findings support the potential use of alternative models for comparative product assessment, but discriminatory performance should not be conflated with the demonstration of bioequivalence qualification.

The broader regulatory environment is increasingly supportive of non-animal and microphysiological approaches [116,117]. The FDA Modernization Act 2.0 removed the statutory requirement that nonclinical testing necessarily include animal studies and explicitly recognized alternatives, including organ chips and other microphysiological systems [118]. This broader policy shift, however, does not itself establish endpoint-specific qualification for dermal pharmacokinetic or IVPT applications [10,12,117,118,119]. For advanced skin platforms, regulatory translation will therefore depend on demonstrating reproducibility, defined acceptance criteria, and performance against appropriate reference methods for the specific context of use [10,12,117,120].

## 8. Fit-for-Purpose Model Selection

The comparative evidence reviewed above indicates that in vitro skin models should not be selected solely on the basis of structural complexity. Their suitability depends on the pharmacokinetic question being addressed, the endpoint measured, the biological process that must be represented, and the intended context of use. Synthetic membranes provide reproducible platforms for formulation screening and passive permeation studies, but cannot reproduce biological tissue retention, metabolism, or physiological clearance. Ex vivo human skin remains the principal conventional reference for human-relevant permeation and tissue retention, whereas reconstructed human skin models provide standardized human-cell-based platforms for comparative testing. Vascularized, bioprinted, and perfused systems further expand the accessible experimental space by introducing architecture-dependent distribution, appendage-like compartments, or dynamic receptor-side removal, but these additional capabilities do not necessarily confer greater quantitative predictive validity [3,6,7,33,34,80,81].

A central implication of this analysis is that an increase in structural complexity should not be interpreted as a monotonic increase in pharmacokinetic relevance. Structural complexity, physiological resemblance, analytical tractability, reproducibility, predictive performance, and regulatory qualification are distinct attributes that do not necessarily covary. Additional biological compartments may broaden the range of mechanisms that can be investigated while simultaneously increasing manufacturing, analytical, and inter-laboratory variability. Conversely, a structurally simpler model with a stable, well-characterized relationship to a reference system may support a more robust quantitative interpretation than a highly complex platform whose performance is insufficiently characterized.

**Figure 5:**
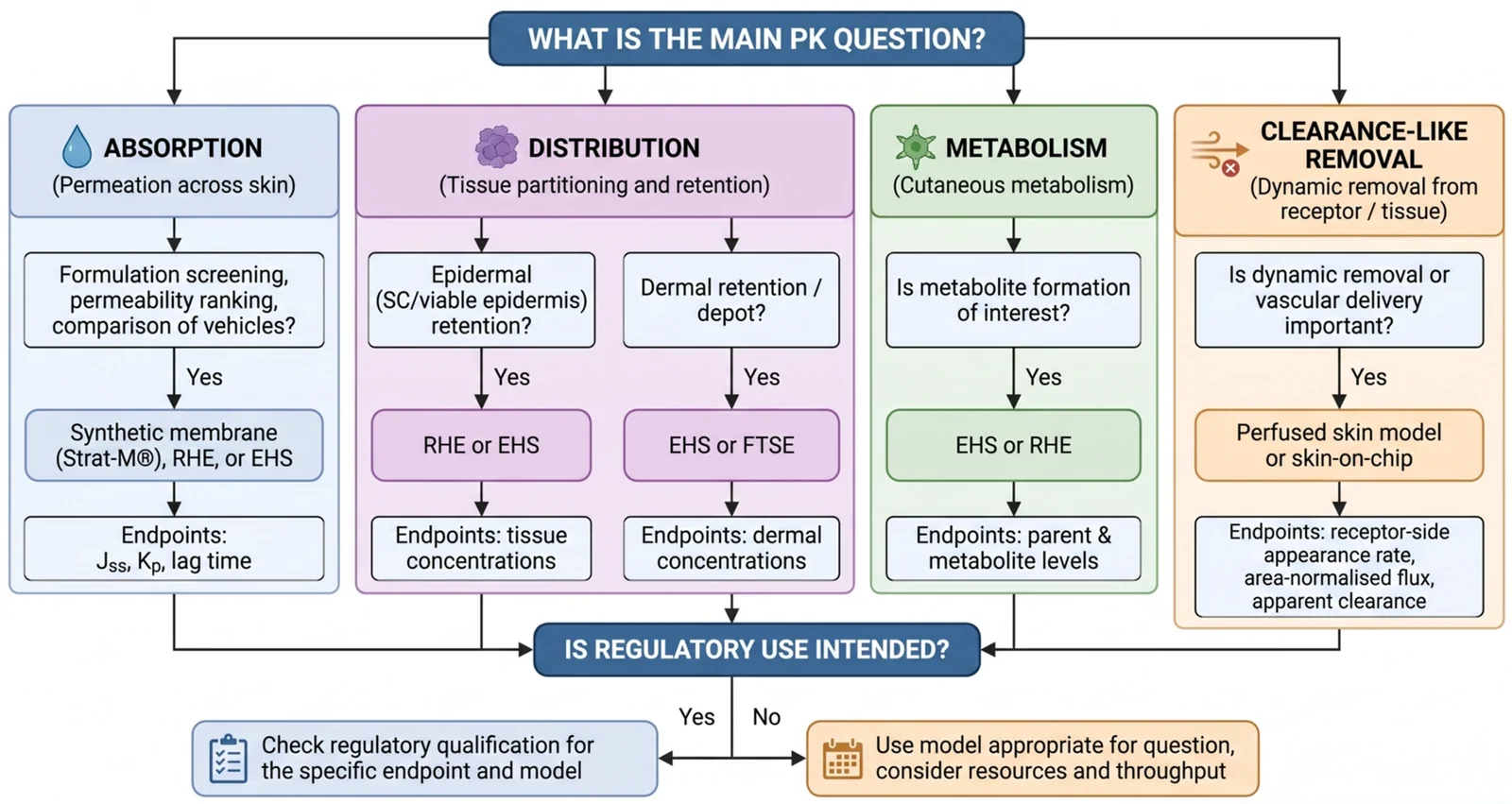
Decision pathway for fit-for-purpose selection of in vitro skin models. Selection proceeds from the pharmacokinetic process under investigation rather than from structural complexity: absorption endpoints (Jss/Kp/tlag) are addressed by synthetic membranes, RHE, or EHS; distribution by RHE or EHS for epidermal retention and EHS or FTSE for dermal retention; metabolism by EHS or RHE; and dynamic removal or vascular delivery by perfused or skin-on-chip systems. A second decision applies to every route: where regulatory use is intended, qualification must be verified for the specific endpoint and context of use, since regulatory acceptance does not transfer between applications. For distribution endpoints, model selection should be complemented by an analytical method appropriate to the spatial question and required resolution, because bulk tissue measurements provide compartment-averaged concentrations, whereas tape stripping, serial-section cutaneous biodistribution, confocal Raman spectroscopy, and mass spectrometry imaging provide depth- or spatially resolved information (Section 4.7).”Decision pathway for fit-for-purpose selection of in vitro skin models”created in BioRender. Zupanic, U. (https://BioRender.com/sbgdd4f) is licensed under CC BY 4.0.

Model selection should therefore be endpoint- and context-specific. Figure 2 illustrates the principal transport pathway and the structural and functional features of each model class, whereas Table 2 summarizes the pharmacokinetic capabilities reported for each platform; these dimensions do not necessarily map onto one another, a central practical consequence of this review. Table 3 translates these capabilities into a practical research-question-driven framework by linking specific applications to the models best suited to address them and by indicating their principal limitations and regulatory status. Figure 5 operationalizes this fit-for-purpose logic as a decision pathway, beginning with the pharmacokinetic question and ending with a check on whether regulatory qualification exists for the intended endpoint and model. A more detailed comparison of structural and dynamic model characteristics is provided in Supplementary Table S2.

Figure 5 and Table 2 and Table 3 indicate which model classes can generate particular pharmacokinetic endpoints, but do not by themselves establish that an endpoint is sufficiently reliable for a given decision. Endpoint suitability should therefore be assessed in terms of repeatability, analytical recovery, and mass balance where applicable, discriminatory ability, robustness, applicability domain, and between-run or between-laboratory reproducibility; claims of human relevance additionally require agreement with an appropriate reference under matched conditions. The required evidence depends on the intended use: formulation ranking primarily requires repeatability, analytical recovery, and discriminatory performance; quantitative human-relevant prediction additionally requires reference agreement within a defined applicability domain; and regulatory use further requires demonstrated robustness, reproducibility, and endpoint-specific qualification.

Together, these comparisons emphasize that no single in vitro skin model fully recapitulates the pharmacokinetic behavior of human skin. Fit-for-purpose selection requires alignment among the intended application, the pharmacokinetic process of interest, the model’s biological and analytical capabilities, and the level of predictive or regulatory evidence required for the resulting data.

## 9. A Research Agenda: What the Field Needs Next

The preceding sections identify a recurring translational limitation: the increasing structural and functional sophistication of in vitro skin models has not yet been matched by a common benchmarking framework that would enable comparison of their pharmacokinetic performance under standardized conditions across platforms and laboratories. Progress, therefore, requires not only further model development but also greater standardization of reference compounds, experimental conditions, reporting practices, and endpoint-specific qualification. Four practical priorities emerge from the evidence reviewed here.

First, future cross-model benchmarking would benefit from a well-defined panel of candidate reference compounds evaluated using a prospectively defined cross-model protocol. Existing cross-model studies rely on a relatively limited set of permeants, although the strength of evidence supporting their use as reference compounds differs. Caffeine and testosterone have the strongest historical basis, including their use in RHE prevalidation and validation programs, whereas salicylic acid and resorcinol provide complementary evidence from matched cross-model comparisons [8,20,46,64]. For the illustrative panel proposed here, compounds were selected according to four criteria: documented use as reference permeants, availability of matched cross-model comparisons, coverage of different physicochemical properties, and demonstrated analytical feasibility in the relevant samples using either radiolabeled material or a validated non-radiolabeled assay [37].

Table 4 presents an illustrative candidate core panel comprising caffeine, salicylic acid, testosterone, and resorcinol. Its purpose is not to establish universal permeability constants, which would be inconsistent with the dose-, formulation-, model-, and method-dependence discussed throughout this review, but to support the generation of compound- and endpoint-specific performance profiles relative to ex vivo human skin under matched conditions.

Such benchmarking would only be informative within a prospectively defined protocol in which critical exposure and method variables are controlled and, whenever scientifically and technically appropriate, matched across models and laboratories. The exposure specification should include the vehicle or formulation composition and, where relevant, pH; test-substance concentration or product strength; applied formulation mass or volume per exposed area and the corresponding active-substance dose per area; finite- or infinite-dose conditions; the application and spreading procedure; exposure duration and total study duration; occlusion conditions; test-surface temperature; and ambient temperature and relative humidity. Test-system variables should include the model and tissue source, donor or batch structure, anatomical site where applicable, tissue thickness, preparation and storage conditions, exposed area, replicate allocation, barrier-integrity method and acceptance criterion, and receptor medium composition, pH, volume, mixing or flow rate, sampling schedule, and evidence of adequate analyte stability, solubility, and sink conditions in the receptor medium [9,10,11,12,37,122].

For retention and distribution endpoints, the end-of-exposure surface-removal procedure should be predefined and reported, including the cleaning materials and medium, the number and sequence of washing or wiping steps, and analyte recovery from all materials used [9,10,11,37]. Any tape-stripping or skin-fractionation procedure should likewise be predefined and consistently applied, and the analytical method should be validated for each matrix in which it is used. The actual applied formulation amount and corresponding active-substance dose, the amount recovered from each relevant compartment, and the overall mass balance should be reported using consistent definitions and prespecified acceptance criteria. When an identical condition is incompatible with a particular platform, the model-specific adaptation should be prospectively defined, scientifically justified, and evaluated for its potential influence on the endpoint rather than assumed to be interchangeable [9,10,11,37].

**Table 4:** Illustrative candidate panel for future cross-model benchmarking and optional mechanistic challenge modules.

Panel Category	Compound or Probe	Approximate logP (Neutral Species)	Primary Property Probed	Rationale	Analytical Feasibility	Key References
Candidate core	Caffeine	−0.07	Small hydrophilic permeant; barrier sensitivity	Established reference permeant used in RHE prevalidation/validation and multiple EHS/alternative-model comparisons; human inter-donor variability has been quantitatively characterized	High-performance liquid chromatography (HPLC) and radiochemical detection have been demonstrated	[8,20,56,64]
Candidate core	Salicylic acid	≈2.3	Ionizable, moderately lipophilic permeant	Provides an ionizable, moderately lipophilic test case; under matched finite-dose conditions, its model rank order differed from that observed for caffeine and testosterone	Radiolabeled quantification has been demonstrated	[64]
Candidate core	Testosterone	≈3.3	Lipophilic permeant; tissue-reservoir behavior	Established reference permeant used in RHE prevalidation/validation; also demonstrates dermal-reservoir effects in full-thickness constructs	HPLC and radiochemical detection have been demonstrated; non-radiolabeled analysis requires consideration of endogenous testosterone	[8,20,60,65,123]
Candidate core	Resorcinol	≈0.8	Small polar phenol	Direct quantitative steady-state flux data are available across Strat-M^®^, EpiSkin^®^, and ex vivo human skin under matched experimental conditions	HPLC quantification has been demonstrated under the conditions of the cited study	[46]
Optional challenge	High-lipophilicity probe (specific compound to be predefined)	>5	Non-polar transport; receptor and device sorption	Extends benchmarking to conditions in which sink limitations and adsorption to materials such as PDMS become important; the probe and receptor conditions would require standardization before inter-laboratory use	Analytical feasibility cannot be assessed until the specific probe is predefined.	[63]
Optional challenge	Metabolically labile ester substrate	Variable	Cutaneous esterase activity	Provides a functional metabolic endpoint based on parent depletion and/or metabolite formation rather than enzyme expression alone; requires validated analytical recovery for the selected substrate	Analytical feasibility cannot be assessed until the specific probe is predefined.	[24]
Optional challenge	Follicular/particle probe	Not applicable	Appendage-associated transport	Relevant only to appendage-bearing models; particle size, charge, surface chemistry, and formulation would require predefined specifications and should therefore be treated as a separate mechanistic module rather than a universal permeability benchmark	Analytical feasibility cannot be assessed until the specific probe is predefined.	[23,81]

Note: The candidate panel is intended to support future cross-model permeation benchmarking, whereas the optional modules address mechanisms that cannot be evaluated using a common small-molecule panel. Its primary qualification objectives are to assess within-laboratory repeatability, rank-order discrimination, and inter-laboratory transferability. Agreement with ex vivo human skin should be evaluated where matched reference data are available, whereas model-specific calibration should be considered a potential downstream application within a defined applicability domain rather than a primary qualification objective. It represents an illustrative framework rather than a consensus recommendation or established standard. Reported analytical feasibility is protocol-, concentration-, and matrix-specific and does not replace validation of the selected method at the intended dose and in all relevant samples [37]. For ionizable compounds such as salicylic acid, donor pH and the corresponding ionization state (logD) should also be reported, because nominal logP alone does not fully characterize the species presented to the barrier.

Second, inter-laboratory transferability should be evaluated systematically for advanced skin models. The strongest established precedent for skin-absorption model validation is the RHE program, in which multicenter prevalidation using caffeine and testosterone was followed by a formal validation study involving nine compounds and ten laboratories, with human epidermis and pig skin serving as reference preparations [8,20]. Comparable multi-laboratory permeation validation has not yet been established for the bioprinted and perfused skin-on-chip platforms evaluated in this review, for which quantitative pharmacokinetic evidence remains predominantly laboratory- and device-specific [6,7,33,34,80].

An initial multi-laboratory transferability study could combine the candidate core panel in Table 4 with harmonized experimental conditions, including predefined dose regimens, receptor conditions, barrier-integrity criteria, analytical recovery, and mandatory mass balance reporting. Finite- and infinite-dose experiments should be treated as separate study arms rather than interchangeable protocols. An infinite-dose arm could characterize steady-state transport under defined boundary conditions, yielding Kp, Jss, and apparent tlag as system-dependent parameters, whereas a finite-dose arm could assess product-relevant cumulative permeation, peak flux (Jpeak, reported together with tpeak), and mass balance [10,12,122]. Such a design would provide initial estimates of intra- and inter-laboratory variability and help identify platform-specific sources of variation. To distinguish the major sources of variability, the study design should preserve the hierarchical structure of the data and separately account for laboratory, operator, experimental run, donor or model batch, replicate, and analytical effects. These components could be estimated using a variance-components or mixed-effects model, with analytical variability assessed through common quality-control samples and, where feasible, replicate or cross-laboratory analyses.

Third, structured data sharing should accompany experimental standardization. Where legally and commercially feasible, de-identified and non-proprietary IVPT datasets should include raw concentration–time data together with essential experimental metadata, including donor or batch structure, dose regimen, receptor medium composition, receptor volume or flow rate, barrier-integrity assessment, analytical recovery, mass balance results, and derived pharmacokinetic endpoints. Consistent reporting of these variables is essential because differences in experimental design can substantially affect the interpretation and comparability of permeation data [8,10,12].

Greater availability of structured experimental datasets would facilitate quantitative cross-study synthesis and provide a broader empirical basis for parameterization and evaluation of mechanistic dermal PBPK and IVIVE models, in which experimentally derived permeability, partitioning, and permeation data can inform predictions of in vivo dermal and systemic exposure [66,67,69,70]. Standardized data structures should therefore complement, rather than replace, standardized experimental protocols.

Fourth, model qualification should be endpoint- and context-of-use-specific rather than treated as a global property of a platform. The evidence reviewed here demonstrates that the same model may be informative for one pharmacokinetic question while remaining inadequate for another. RHE may support comparative formulation discrimination under matched conditions, while remaining unsuitable for uncalibrated prediction of absolute human permeability [20,79]. FTSE can provide information on dermal retention but does not recapitulate vascular clearance, whereas perfused skin-on-chip systems can improve receptor-side boundary conditions without fully reproducing the physiology of dermal blood and lymphatic removal. Qualification should therefore specify the model, pharmacokinetic endpoint, dose regimen, analytical readout, performance criteria, and intended context of use. Such a fit-for-purpose approach would allow evidence to accumulate incrementally for defined applications without implying universal equivalence to human skin [33,65].

Together, these priorities shift the emphasis from model novelty or structural complexity toward reproducible performance within a defined pharmacokinetic context. The candidate framework in Table 4 provides one possible basis for cross-model benchmarking, while harmonized experimental protocols, structured reporting, and inter-laboratory evaluation would provide the comparability and variance estimates needed to determine whether an advanced model is sufficiently reproducible and transferable for a defined endpoint and context of use.

## 10. Limitations of This Review

This review was designed as a critical narrative review rather than a systematic review, and no formal risk-of-bias assessment or meta-analysis was undertaken. Although the literature search was structured across multiple databases using predefined search concepts and eligibility criteria, the evidence was synthesized narratively and should not be interpreted as an exhaustive systematic assessment. Quantitative comparisons across studies are further constrained by substantial heterogeneity in permeants, formulations, dose regimens, tissue preparation, receptor conditions, sampling schedules, and analytical recovery; accordingly, the quantitative values discussed in this review should be interpreted in the context of the experimental conditions under which they were obtained.

The evidence base is also uneven across pharmacokinetic processes and model classes. Direct matched comparisons are considerably more abundant for absorption than for cutaneous distribution, metabolism, or clearance-like removal, while quantitative benchmarking and inter-laboratory validation of advanced bioprinted, vascularized, and microphysiological platforms remain limited. Finally, regulatory acceptance is endpoint-, context-of-use-, and jurisdiction-specific and may evolve as new qualification evidence emerges. The model comparisons and fit-for-purpose framework presented here should therefore be interpreted as an evidence-informed synthesis of current capabilities and limitations rather than as a universal ranking of model performance.

## 11. Translational Outlook and Conclusion

No current in vitro system fully recapitulates the pharmacokinetic behavior of human skin. Model relevance is therefore inherently endpoint- and context-specific and should be distinguished from structural complexity. Biological capability, analytical performance, predictive agreement with reference tissue, reproducibility, and regulatory qualification represent distinct dimensions of model performance, and strength in one does not imply strength in the others. Increasing model complexity should consequently not be assumed to confer greater pharmacokinetic or predictive validity.

Ex vivo human skin remains the principal conventional reference for human-relevant permeation, tissue retention, and mass balance assessment because it preserves native barrier architecture and is compatible with established IVPT and OECD TG 428-based workflows [2,9,13]. Its limitations, including restricted availability, donor and anatomical-site variability, storage sensitivity, and absence of active vascular or lymphatic clearance, create complementary roles for alternative models [2,56]. Synthetic membranes provide reproducible platforms for passive permeation screening and formulation ranking; porcine skin offers a practical biological comparator when human tissue is unavailable; and RHE and FTSE provide standardized human-cell-based systems for comparative permeation, local distribution, and selected metabolic endpoints. Bioprinted, vascularized, and perfused platforms further extend the experimental space toward spatially defined tissue architecture and dynamic downstream removal, although quantitative benchmarking and inter-laboratory validation remain limited [3,6,7,33,34,80,81].

Importantly, the presence of a biological feature should not be equated with demonstrated pharmacokinetic function. For metabolism-sensitive compounds, metabolic competence requires functional evidence of pathway-relevant activity and metabolite formation rather than inference from cellular viability or enzyme expression alone [24,25,28,29]. Similarly, vascular-like structures or continuous perfusion can introduce dynamic downstream removal and improve receptor-side control, but should not be interpreted as reproducing physiological dermal vascular or lymphatic clearance without appropriate functional validation [6,33,34].

Future progress will therefore depend less on increasing model complexity per se than on improving model characterization and endpoint-specific qualification. Matched benchmarking against appropriate reference tissues, harmonized experimental conditions and reporting, functional validation of relevant biological processes, inter-laboratory transferability, and mechanistic IVIVE/PBPK modeling will be important for translating model-specific measurements toward human exposure. The evidence reviewed here does not currently support a single universal scaling relationship between alternative barriers and human skin, although calibration within a prospectively defined compound, formulation, and experimental applicability domain may remain possible. Model selection should therefore be driven by the pharmacokinetic process of interest, the biological capabilities required to represent it, the measured analytical endpoints, and the intended context of use. Where a broader characterization of cutaneous disposition is required, complementary models and analytical readouts may therefore be combined, whereas a single fit-for-purpose model may be sufficient for a narrowly defined question.

Ultimately, the most informative in vitro skin model is not necessarily the most complex, but the one whose performance is sufficiently characterized for the specific pharmacokinetic question it is intended to address.

## Figures and Tables

**Table 1:** Major methodological sources of bias in in vitro skin permeation studies.

Source of Bias	Mechanism/Setting	Expected Effect on Measured Data	Key Control
Receptor-side limitations	Inadequate receptor solubilization, loss of sink conditions, or an unstirred water layer can add receptor-side resistance. Receptor medium composition alters Franz-cell performance, and resistance has been demonstrated experimentally [32,33].	Can ↓ apparent J and Kp when receptor-side transport contributes materially to total resistance.	Verify receptor solubility and sink conditions; standardize receptor composition, stirring, and geometry; consider flow-through conditions when justified.
Compromised barrier integrity	Mechanical damage, inappropriate handling, or other insults can reduce stratum corneum barrier function. Electrical resistance is inversely associated with water permeability, and the FDA recommends excluding sections with compromised integrity [10,49].	Usually ↑ apparent permeability/absorption.	Apply a predefined integrity test and acceptance criterion before dosing; standardize tissue preparation and handling.
Donor-side changes or dose-regimen mismatch	Finite-dose evaporation, dose depletion, and precipitation/crystallization can change the donor boundary condition and the shape of the permeation profile; finite- and infinite-dose experiments are therefore not directly interchangeable [10,36].	↕ Context-dependent; no universal directional bias.	Match the dosing regimen to the research question; report dose per area, vehicle, and occlusion conditions, and characterize relevant donor-side changes.
Material interactions	PDMS can sorb lipophilic compounds and reduce freely available concentrations; some vehicles can also alter the properties of synthetic membranes [41,55].	↕ Context-dependent: PDMS sorption may decrease apparent recovery and transport, whereas membrane–vehicle interactions may increase or decrease apparent permeation.	Perform material-recovery and membrane–vehicle compatibility controls.
Incomplete analytical recovery	Incomplete quantification across receptor fluid, skin, and other test-system compartments compromises dose recovery and mass balance [9,37].	↓ quantified recovery in the affected compartment, and prevents unambiguous interpretation of mass balance.	Quantify relevant test-system compartments and report overall recovery; validate analytical recovery where appropriate.
Analytical quantification approach	Radiolabeled measurements depend on radiochemical purity and label position/stability; exchangeable tritium labels or transformation products may complicate interpretation. Non-radiolabeled measurements depend on method validation, extraction recovery, stability, and liquid chromatography–mass spectrometry (LC–MS) matrix effects [9,37,60]	↕ Context-dependent; may bias compartment-specific amounts or apparent recovery.	Radiolabeled: document radiochemical purity, label position/stability, and apparatus recovery. Non-radiolabeled: validate selectivity, accuracy, precision, stability, extraction recovery, and LC–MS matrix effects.
Ignoring donor-level data structure	Multiple skin sections from the same donor are within-donor replicates. FDA IVPT analysis explicitly distinguishes replicate-level, within-donor, and inter-donor information [10,57].	No fixed ↑/↓, incorrect treatment of donor structure can distort variance estimation and statistical inference.	Preserve donor identity; use matched within-donor treatment allocation and account explicitly for within- and between-donor variability.

Note. **↑** and **↓** indicate the expected direction of the measured endpoint under the stated mechanism; ↕ indicates that the direction depends on the permeant, formulation, or experimental conditions. These directions are mechanistic expectations and should not be interpreted as numerical correction factors.

**Table 2:** Reported functional pharmacokinetic capabilities of in vitro skin models across cutaneous disposition processes.

Model	Absorption	Distribution/Retention	Metabolism	Clearance-Like Removal	Key References
PDMS/silicone	Measures passive diffusion and formulation release; useful for device validation, but limited physiological prediction	No biological distribution; retention reflects polymer partitioning	None	No intrinsic clearance; receptor-side removal depends on static receptor conditions or imposed flow	[2,33,63]
Strat-M^®^	Reproducible passive permeation; useful for formulation ranking under controlled conditions; predictive value is compound-, vehicle-, and dose-dependent	No biological distribution; retained drug reflects recovery from an artificial membrane, not stratum corneum, epidermal, or dermal deposition	None	No vascular or lymphatic clearance; receptor phase only	[45,55,62]
Ex vivo human skin-EHS	Closest conventional reference for human skin absorption; supports Kp, Jss, tlag, and receptor-phase permeation	Native layer architecture enables tape stripping, epidermis/dermis separation, and tissue-retention analysis	Fresh tissue may retain native cutaneous xenobiotic-metabolizing activity; activity declines with handling, storage, and loss of viability	No active vascular or lymphatic clearance; the receptor phase is an artificial sink	[2,9,24,26,32,54,56,64,99]
Ex vivo pig skin	Useful animal surrogate for comparative permeation and formulation rank-order; not quantitatively interchangeable with EHS	Supports tape stripping and tissue sectioning; layer-specific retention may differ from human skin	May retain xenobiotic-metabolizing activity, but enzyme expression and activity are species-dependent	No active clearance; receptor phase only	[2,4,9,71,72,75,76]
Ex vivo rat/mouse/rabbit skin	Often overestimates human absorption; useful mainly for mechanistic or conservative screening	Native animal layers are present, but quantitative extrapolation to human skin is uncertain	Active but species-specific xenobiotic-metabolizing profile	No active clearance; receptor phase only	[2,71,72,78,121]
RHE (EpiDerm^®^, EpiSkin^®^, SkinEthic^®^)	Useful for comparative permeation and rank-order screening; frequently more permeable than native reference tissues, although the magnitude and direction of deviation are compound- and model-dependent	Captures stratum-corneum-like and viable epidermal uptake only; cannot model dermal retention or vascular distribution	Viable human cells; selected phase II, non-CYP and esterase-related pathways partly preserved; weak basal CYP activity	No vascular clearance; receptor phase only	[5,20,24,28,79,102,103]
LSE/FTSE (Phenion^®^ FT, EpiDermFT^®^, LabSkin)	Supports permeation across epidermal and dermal-equivalent compartments; dermal partitioning may delay receptor-phase appearance, although barrier permeability often remains higher than in native skin	Enables epidermis–dermis partitioning and dermal retention; dermis can act as a reservoir for lipophilic compounds	Viable cells; phase II, non-CYP and esterase-related pathways partly preserved; weak basal CYP activity	No active clearance; static dermis can retain the drug but cannot remove it through blood flow	[2,5,24,28,29,65]
Vascularized full-thickness constructs	Proof-of-concept topical-to-vascular and vascular-to-tissue transport has been demonstrated in a perfusable vascularized construct produced by molding, self-assembly, and microfluidic integration rather than bioprinting; quantitative benchmarking remains limited	Supports compartment-resolved dermal and vascular-like distribution and, in perfused designs, bidirectional topical-to-vascular and vascular-to-tissue transport	Depends on the incorporation of viable cell populations and the construct design; compound-specific metabolic validation remains limited	Perfusion can provide dynamic clearance-like removal, but does not reproduce full physiological blood or lymphatic clearance	[7]
3D-bioprinted skin constructs	Quantitative permeation datasets remain sparse; standardized Kp, Jss, and lag-time comparisons with ex vivo human skin are lacking among the studies identified in this review	Customizable architecture can incorporate dermal and appendage-like compartments, providing a basis for mechanistic distribution studies; quantitative follicular transport remains insufficiently validated	Not well characterized as a model class; metabolic relevance depends on construct design and requires endpoint-specific validation	Non-perfused constructs behave as static reservoirs; bioprinted constructs can be perfused when vascular channels are incorporated, but clearance-specific validation remains limited	[2,3,80,81]
Perfused/microfluidic skin-on-chip	Dynamic flow can improve sink control and reduce unstirred-water-layer artifacts; the benefit is model- and compound-dependent	Enables time-resolved barrier-to-receptor transport; vascular-like distribution only in perfused and/or vascularized designs	Depends on incorporated viable skin; isolated skin-only chips remain weakly validated for metabolite profiling	Continuous perfusion provides receptor-side removal but does not reproduce full dermal blood/lymph clearance	[6,33,34,35,80]

Note: Table 2 summarizes reported functional pharmacokinetic capabilities and should not be interpreted as a measure of structural complexity, quantitative agreement with ex vivo human skin, reproducibility, evidence maturity, or regulatory qualification. Structural representation is shown separately in Figure 2, whereas comparative evidence, reproducibility, evidence maturity, and regulatory context are distinguished in Table 3.

**Table 3:** Fit-for-purpose selection of in vitro skin models according to the pharmacokinetic research question.

Research Question	Best-Fit Model(s)	Regulatory Status	Demonstrated Function	Evidence Status	Main Limitations	Key References
Early formulation ranking/high-throughput screening	Strat-M^®^ or other appropriately characterized synthetic membranes	Industrial screening tool; not OECD-validated as a human skin substitute	Comparative permeation and formulation rank ordering under controlled conditions.	Ref.: EHS comparisons reported; agreement is compound-, vehicle-, and dose-dependent. Rep.: high under controlled conditions. Maturity: comparative evidence; no formal pharmacokinetic qualification.	No viable compartments; cannot assess tissue retention, metabolism, or clearance; high-polyol or infinite-dose conditions may disrupt performance	[45,46,47,48,55,61]
Human-reference permeation/quantitative IVPT	Ex vivo human skin; porcine skin for comparative IVPT when EHS is unavailable	OECD TG 428-compatible reference tissue	Supports Kp, Jss, tlag, receptor-phase permeation, tape stripping, tissue retention, and comparative rank-order assessment.	Ref.: EHS is the principal conventional reference; porcine skin is not quantitatively interchangeable with EHS. Rep.: donor-, site-, and handling-dependent. Maturity: established conventional reference.	Limited availability, high variability, and storage sensitivity; frozen skin may alter barrier properties; no active vascular/lymphatic clearance	[4,9,56,75,76,77]
Regulatory hazard testing (skin irritation/corrosion)	Validated RHE models	OECD TG 439 for irritation; OECD TG 431 for corrosion	Validated assessment of skin irritation and corrosion.	Ref.: hazard validation does not establish agreement for pharmacokinetic endpoints. Rep.: demonstrated in formal validation programs. Maturity: formally validated for irritation/corrosion.	Immature SC lipid/protein organization; often higher permeability than EHS; no dermis, appendages, vasculature, or clearance	[5,8,20,114,115]
Comparative formulation discrimination/epidermal targeting	RHE for exploratory or comparative product discrimination; EHS remains the reference for regulatory IVPT	Research/comparative pharmacokinetic use; not OECD-validated for absorption	Formulation ranking, profile matching, and discrimination of selected topical formulations under matched conditions.	Ref.: selected comparisons with EHS reported; absolute human prediction is not established. Rep.: primarily within-laboratory and model-dependent. Maturity: comparative evidence; no formal absorption qualification.	Lacks dermal reservoir and vascular clearance; epidermal uptake may be biased by overpermeability	[5,8,20,79]
Dermal retention/epidermis–dermis partitioning	EHS for native reference; FTSE/LSE for controlled human-cell-based comparative studies	EHS compatible with OECD TG 428-based dermal absorption studies; FTSE/LSE remain research/comparative PK platforms, not OECD-qualified for quantitative dermal absorption	Layer-specific recovery, epidermis–dermis partitioning, and dermal reservoir assessment.	Ref.: EHS is the native reference; quantitative benchmarking of FTSE/LSE against EHS remains limited. Rep.: EHS is donor- and site-dependent; engineered models remain model-dependent. Maturity: EHS established; FTSE/LSE mainly comparative/research-stage.	EHS is donor-, site-, and handling-dependent and lacks active vascular/lymphatic clearance after excision; FTSE/LSE show non-native barrier organization and lack physiological vascular removal	[2,5,9,11,54,65]
Appendage-associated or vascular-like distribution	Bioprinted appendage-containing constructs for appendage-associated localization; vascularized/perfused full-thickness constructs for vascular-like distribution and bidirectional transport	Research-stage platforms; no current OECD qualification for quantitative dermal absorption, appendage-associated transport, or vascular clearance	Topical-to-vascular and vascular-to-tissue transport, as well as appendage-associated localization, have been demonstrated in selected constructs.	Ref.: limited. Rep.: limited. Maturity: proof-of-concept/research-stage.	Architectural representation does not establish a validated pharmacokinetic function; quantitative benchmarks for follicular uptake, transfollicular transport, vascular recovery, and clearance remain sparse; technical complexity remains a significant limitation.	[7,23,80,81]
Clearance-sensitive permeation/dynamic receptor-side transport	Perfused or microfluidic skin-on-chip	Research-stage; no current OECD acceptance	Continuous receptor renewal, improved sink control, reduced unstirred-water-layer artifacts, and time-resolved sampling.	Ref.: limited. Rep.: limited inter-laboratory evidence. Maturity: research-stage.	Does not fully reproduce physiological dermal blood/lymph clearance; performance depends on chip geometry, flow rate, barrier type, and material adsorption	[2,6,33,34,35,41,42]
Dermal absorption + systemic metabolism	Coupled skin–liver microphysiological system/Chip2 systems	Research-stage; no current OECD acceptance	Coupled skin permeation and downstream hepatic biotransformation.	Ref.: limited. Rep.: limited. Maturity: research-stage.	Not a skin-only model; technically complex, low-throughput, and model-dependent	[104,105]

Note: Structural representation is presented separately in Figure 2. In Table 3, demonstrated function is distinguished from matched reference comparison (Ref.), reproducibility (Rep.), evidence maturity, and regulatory status because these attributes do not necessarily covary. “Limited” indicates that the corresponding evidence remains sparse or insufficiently established in the literature reviewed and should not be interpreted as evidence of the absence of the capability.

## Data Availability

No new data were created or analyzed in this study. Data sharing is not applicable to this article.

## Supplementary Materials

The following supporting information can be downloaded at https://www.mdpi.com/article/10.3390/pharmaceutics18091152/s1. Table S1: Barrier integrity tests and reported acceptance criteria for ex vivo skin; Table S2: Structural and dynamic characteristics, limitations, and validation status of in vitro skin models.

## Abbreviations

The following abbreviations are used in this manuscript:

ADH	alcohol dehydrogenase
ADME	absorption, distribution, metabolism, and excretion (in this review, the fourth process is treated as clearance-like removal, since in vitro skin models have no excretory function)
AHT	4-amino-2-hydroxytoluene
AMT	total cumulative amount permeated
BCRP	breast cancer resistance protein
CBM	cutaneous biodistribution method
COMT	catechol-O-methyltransferase
CRS	confocal Raman spectroscopy
CYP	cytochrome P450
dOFM	dermal open-flow microperfusion
EFSA	European Food Safety Authority
EHS	ex vivo human skin
EMA	European Medicines Agency
ER	electrical resistance
FDA	U.S. Food and Drug Administration
FMO	flavin-containing monooxygenase
FTSE	full-thickness skin equivalent
GelMA	gelatin methacryloyl
GST	glutathione S-transferase
HPLC	high-performance liquid chromatography
IVIVE	in vitro–in vivo extrapolation
IVPT	in vitro permeation testing
IVRT	in vitro release testing
LC–MS	liquid chromatography–mass spectrometry
LSE	living skin equivalent
MALDI	matrix-assisted laser desorption/ionization
MIVO	multi-in-vitro-organ (microphysiological platform)
MPS	microphysiological system
MRP	multidrug resistance-associated protein
MSI	mass spectrometry imaging
NAT	N-acetyltransferase
OECD	Organisation for Economic Co-operation and Development
PBPK	physiologically based pharmacokinetic
PDMS	poly(dimethylsiloxane)
P-gp	P-glycoprotein
RHE	reconstructed human epidermis
SC	stratum corneum
TEWL	transepidermal water loss
TG	Test Guideline
UGT	UDP-glucuronosyltransferase
UHPLC–MS/MS	ultra-high-performance liquid chromatography–tandem mass spectrometry
XME	xenobiotic-metabolizing enzyme
A	exposed diffusion area
Cd	dissolved donor concentration in contact with the barrier
Cin, Cout	compound concentration in the inlet and outlet perfusate
CLapp	apparent concentration-normalized clearance
Cref	concentration in the compartment from which the compound is removed
Deff	effective diffusion coefficient
Jmax	maximum flux
Jpeak	peak flux under finite-dose conditions
Jss	steady-state flux
Kp	apparent permeability coefficient
Q(t)	cumulative amount permeated per unit area
Qf	volumetric flow rate
Sv	saturation solubility of the compound in the applied vehicle
tlag	lag time
tpeak	time of peak flux
