# Supplementary material for: Pharmacokinetic Relevance of In Vitro Skin Models: Absorption, Cutaneous Distribution, Metabolism, and Clearance-like Removal in Dermal and Transdermal Drug Testing
Source: Pharmaceutics. 2026 Sep 14;18(9):1152. doi: 10.3390/pharmaceutics18091152 (PMC13610528; doi:10.3390/pharmaceutics18091152)
Supplement: Supplementary file 1 [file pharmaceutics-18-01152-s001.zip › pharmaceutics-4539217-supplementary.pdf]

SUPPLEMENTARY MATERIAL

Pharmacokinetic Relevance of *In Vitro* Skin Models: Absorption, Cutaneous Distribution, Metabolism, and Clearance-Like Removal in Dermal and Transdermal Drug Testing

Filip Dugonik[1,♯], Urška Jeršič[1,♯], Lejla Kač[1,♯], Uroš Maver[1,2,*], Tina Maver[1,2,*]

[1] Institute of Biomedical Sciences, Faculty of Medicine, University of Maribor, Taborska ulica 8, 2000 Maribor, Slovenia; filip.dugonik@student.um.si; urska.jersic@student.um.si; lejla.kac@student.um.si

[2] Department of Pharmacology, Faculty of Medicine, University of Maribor, Taborska ulica 8, 2000 Maribor, Slovenia; uros.maver@um.si; tina.maver@um.si

♯ These authors contributed equally to the article and share the first authorship.

* Correspondence: tina.maver@um.si, uros.maver@um.si; Tel.: 00386 2 2345 839

<u>SUPPLEMENTARY MATERIAL</u>

Table S1. Barrier integrity tests and reported acceptance criteria for *ex vivo* skin.

Test	What it measures	Reported criteria for intact human skin	Main limitations	Ref
**Transepidermal water loss (TEWL)**	Evaporative water loss across the skin surface	≤ approximately 10 g/m²/h in one comparative study; 0.7–5 g/m²/h in a large multi-chemical dataset based on laboratory-historical acceptance values	Measures water loss rather than permeability of the test compound; sensitive to ambient humidity, airflow, and measurement conditions; acceptance criteria are often based on laboratory experience rather than direct validation against compound transport	[36, 51]
**Electrical resistance (ER)**	Ionic conductance across the skin barrier, predominantly reflecting stratum corneum integrity	≥ 2 kΩ in one comparative study; ≥ 10 kΩ for a 2.54 cm² exposed area (25.4 kΩ × cm²); ≥ 23 kΩ for 0.64 cm² (14.7 kΩ × cm²); ≥ 5 kΩ for 2.54 cm² (12.7 kΩ × cm²) proposed following retrospective analysis	Strongly dependent on exposed area, electrode configuration, medium, temperature, and instrumentation; overly stringent thresholds may reject samples that otherwise show acceptable barrier function	[49-52]
**Tritiated water permeability**	Permeability to a radiolabeled hydrophilic probe, primarily reflecting polar-pathway barrier integrity	Reported Kp thresholds range from $\leq 1.5 \times 10^{-3}$ to $\leq 4.5 \times 10^{-3}$ cm/h, depending on study and protocol. Regulatory guidance may apply alternative acceptance criteria based on absorbed amount rather than Kp	Requires handling of radiolabeled material; no universally harmonized threshold; agreement with the permeability of lipophilic probes may be limited	[10, 49-53]
**Lipophilic probe (e.g., radiolabeled octanol)**	Permeability of a lipophilic probe through non-polar transport pathways	No harmonized acceptance threshold established	Not established as a routine stand-alone integrity test; interpretation is probe-dependent, but it may provide complementary information not captured by water-based tests	[53]
**Visual and histological inspection**	Macroscopic or microscopic evidence of tissue damage, preparation artefacts, and structural abnormalities	No quantitative acceptance criterion	Insensitive to subtle functional barrier defects; primarily complementary to quantitative integrity measurements	[9]

Table note. Reported acceptance criteria vary with experimental setup, exposed area, instrumentation, and laboratory-specific procedures and should therefore not be interpreted as universal cut-offs. Where applicable, electrical resistance values are reported together with area-normalized values to facilitate comparison across diffusion-cell geometries.

SUPPLEMENTARY MATERIAL

Table S2. Structural and dynamic characteristics, limitations, and validation status of *in vitro* skin models.

Model	Structural and dynamic features	Principal limitations	Regulatory / validation status	Key references
**PDMS / silicone**	Hydrophobic synthetic polymer membrane; non-viable; used in static diffusion or as a reference barrier in microfluidic devices	Hydrophobic polymer affinity can distort apparent permeation, especially for lipophilic compounds	No regulatory acceptance as a human skin substitute	[2, 33, 63]
**Strat-M®**	Multilayer, lipid-containing synthetic membrane; non-viable; usually used in static Franz-cell IVPT	Cannot assess tissue retention, metabolism, or clearance; deviation from human skin permeation is compound- and vehicle-dependent without a consistent direction of bias; lacking organized lamellar lipids, it cannot predict enhancers acting by lipid perturbation; performance is affected by infinite-dose and aggressive or high-polyol vehicles	Industrial screening tool; not OECD-validated as a human skin substitute	[45-48, 55, 61, 62]
***Ex vivo* human skin (EHS)**	Native human stratum corneum, viable epidermis, and dermis; appendageal structures may be partly preserved; standard use is static Franz-cell IVPT	Limited supply, high cost, donor/site/storage variability; reported freeze–thaw effects on barrier function are compound- and protocol-dependent, with the most direct evidence from animal tissue and limited human-specific data (Section 3.2)	OECD TG 428-compatible reference tissue	[2, 9, 54, 56, 64]
***Ex vivo* pig skin**	Full-thickness animal skin; closer to human skin than most rodent/rabbit models; static Franz-cell IVPT	Species, anatomical site, thickness, and storage effects remain; they may overestimate human permeation for some highly lipophilic xenobiotics	TG 428-compatible animal comparator when EHS is unavailable	[2, 4, 9, 71, 72, 75, 76]
***Ex vivo* rat/mouse/rabbit skin**	Full-thickness non-porcine animal skin; generally thinner and more permeable than human skin; species-dependent follicles and lipid organization	Limited value for quantitative human translation when EHS or pig skin is available	Preclinical/research use; no regulatory equivalence to human skin	[2, 71, 72, 78, 121]
**RHE (EpiDerm®, EpiSkin®, SkinEthic®)**	Stratified human keratinocyte epidermis with stratum-corneum-like surface; no dermis, appendages or vasculature; static insert/Franz format	Immature stratum corneum lipid/protein organization; no dermis; unsuitable for uncalibrated absolute permeation prediction	OECD TG 439/431 validated for irritation/corrosion; PK use mainly comparative/research	[5, 20, 24, 28, 79, 102, 103]
**LSE / FTSE (*Phenion® FT, EpiDermFT®, LabSkin*)**	Stratified epidermis plus fibroblast-populated dermal equivalent; usually no appendages or perfused vasculature; static format	Incomplete native barrier maturation; no vascular clearance; not standardized for quantitative absorption	Research/comparative PK platform; not OECD-validated for absorption or clearance	[2, 5, 24, 28, 29, 65]
**Vascularized full-thickness constructs**	Human-cell-based full-thickness constructs containing perfusable vascular-like channels or networks may be produced by molding, self-assembly, and microfluidic integration without bioprinting.	High technical complexity; sparse quantitative benchmarking; incomplete validation against *ex vivo* human skin.	Research-stage platform; no OECD regulatory validation for quantitative dermal absorption.	[7]
**3D-bioprinted skin constructs**	Spatially patterned human-cell-based constructs produced by additive deposition of cells and matrix may incorporate keratinocytes, fibroblasts,	High technical complexity; incomplete barrier maturation; limited reproducibility and sparse standardized Kp/Jss/lag-time datasets.	Research-stage platform; no OECD regulatory validation.	[3, 80, 81]

	endothelial cells, melanocytes, or appendage-associated cells.			
**Perfused / microfluidic skin-on-chip**	Platform-dependent; may integrate synthetic membranes, *ex vivo* skin, RHE/FTSE, or bioprinted skin; continuous receptor-side flow	Sensitive to device geometry, flow rate, barrier selection, and material adsorption; limited inter-laboratory validation	Advanced research platform; no current OECD acceptance	[6, 33-35, 80]
